# Neutral and Adaptive Drivers of Microgeographic Genetic Divergence within Continuous Populations: The Case of the Neotropical Tree *Eperua falcata* (Aubl.)

**DOI:** 10.1371/journal.pone.0121394

**Published:** 2015-03-25

**Authors:** Louise Brousseau, Matthieu Foll, Caroline Scotti-Saintagne, Ivan Scotti

**Affiliations:** 1 INRA, UMR745 EcoFoG Ecologie des forêts de Guyane, Campus Agronomique BP316, 97379 Kourou Cedex, France; 2 INRA—Université de Lorraine, UMR1137 EEF Ecologie et Ecophysiologie Forestière, allée de l’Arboretum, 54280 Champenoux, France; 3 INRA, UR629 URFM Ecologie des Forêts Méditerranéennes, Domaine Saint Paul, Site Agroparc CS 40509, 84914 Avignon Cedex 9, France; 4 School of Life Sciences, Ecole Polytechnique Fédérale de Lausanne (EPFL), Station 15, CH-1015 Lausanne, Switzerland; 5 Swiss Institute of Bioinformatics, Lausanne, Switzerland; Aristotle University of Thessaloniki, GREECE

## Abstract

**Background:**

In wild plant populations, genetic divergence within continuous stands is common, sometimes at very short geographical scales. While restrictions to gene flow combined with local inbreeding and genetic drift may cause neutral differentiation among subpopulations, microgeographical variations in environmental conditions can drive adaptive divergence through natural selection at some targeted loci. Such phenomena have recurrently been observed in plant populations occurring across sharp environmental boundaries, but the interplay between selective processes and neutral genetic divergence has seldom been studied.

**Methods:**

We assessed the extent of within-stand neutral and environmentally-driven divergence in the Neotropical tree *Eperua falcate* Aubl. (Fabaceae) through a genome-scan approach. Populations of this species grow in dense stands that cross the boundaries between starkly contrasting habitats. Within-stand phenotypic and candidate-gene divergence have already been proven, making this species a suitable model for the study of genome-wide microgeographic divergence. Thirty trees from each of two habitats (seasonally flooded swamps and well-drained plateaus) in two separate populations were genotyped using thousands of AFLPs markers. To avoid genotyping errors and increase marker reliability, each sample was genotyped twice and submitted to a rigorous procedure for data cleaning, which resulted in 1196 reliable and reproducible markers.

**Results:**

Despite the short spatial distances, we detected within-populations genetic divergence, probably caused by neutral processes, such as restrictions in gene flow. Moreover, habitat-structured subpopulations belonging to otherwise continuous stands also diverge in relation to environmental variability and habitat patchiness: we detected convincing evidence of divergent selection at the genome-wide level and for a fraction of the analyzed loci (comprised between 0.25% and 1.6%). Simulations showed that the levels of differentiation for these outliers are compatible with scenarios of strong divergent selection.

## Introduction

Microgeographic genetic divergence [1, 2] (i.e. the genetic divergence occurring within continuous populations over geographical scales in the same range as species’ dispersal neighborhood, in spite of extensive gene flow) has been frequently demonstrated in plant populations, at least as early as the middle of the 20th century for both phenotypic traits [3–5] and molecular markers [1, 6–9]. Microgeographic divergence has been the subject of major review articles [10–12] arguing that adaptive processes are relatively widespread at these very local scales. However, Spatial Genetic Structure (SGS) is also common at local scales in wild plant populations [13, 14]. This commonly implies neutral divergence caused by restrictions in gene flow (pollen and seeds), genetic drift and mating processes (such as mating among neighbors and local inbreeding) [15, 16]. These processes are supposedly reinforced in plants because they are sessile, even more in trees because of their long life cycle and large progeny sizes [17–19]. Microgeographic neutral divergence is very common in tropical tree species [13, 20, 21], although the observed genetic structures are generally shallow. Pollen and seed flow are often restricted because air humidity and frequent precipitation prevent wind dispersion of pollen and seeds, and because the heavy seeds are often dispersed by gravity close to maternal tree crowns [16, 22], as it is the case in the bat-pollinated and autochorous canopy tree *Eperua falcata* Aublet (Fabaceae). Consequently, mating among neighbors is frequent in aggregative tree species, causing local inbreeding and contributing to the spatial genetic structuring [20].

While neutral genetic divergence is independent of habitat variation (except when such variation induces barriers to dispersal [23]), adaptive genetic divergence is driven by habitat transitions at least for some specific loci [8, 10, 23]. In this case, the genetic differentiation is expected to be stronger for adaptive loci than for neutral ones. This difference provides a theoretical framework for the discrimination of neutral and adaptive sources of microgeographic differentiation.

Amazonian lowland forests are characterized by complex habitat patchiness whereby environmental conditions vary at a small spatial scale (i.e. in the order of hundreds of meters). The succession of waterlogged bottomlands and well-drained ‘*terra firme*’ plateaus is associated with strong variations in tree communities [24–27]. Microgeographic environmental variability is thus likely to participate to the maintenance of high diversity of tree species in the forest landscapes of Amazonia [28]. More precisely, it has been suggested that divergent selective pressures among local habitat types may have driven the specialization of trees species for local conditions, and that ecological divergences among congeneric species would result from adaptive radiations along topography gradients [29]. In *E*. *falcata*, a recent study has revealed footprints of divergent selection between local subpopulations occupying distinct habitats at stress-response genes [8]. Genetic differentiation was accompanied by consistent phenotypic divergence for growth and leaf physiology at the seedling stage in *E*. *falcata* and in the congeneric *E*. *grandiflora* [30]. These preliminary results make *E*. *falcata* a good model for the analysis of adaptive processes over microgeographical scales.

In this study, we analyzed the neutral and adaptive sources of genetic structuring within continuous stands of *E*. *falcata* in the eastern Guiana shield (French Guiana). To achieve our goals, four populations (corresponding to the replication of the microgeographic ‘hilltop *versus* bottomland’ environmental contrast in two distinct stands) were scanned with approximately 1200 AFLP markers. Genome-wide spatial genetic structure was evaluated and the extent of genetic divergence was assessed at both regional and microgeographical scales. A landscape approach was combined with outlier detection tests to distinguish between neutral and adaptive sources of genetic divergence, and to determine whether microgeographic adaptation to local habitat patchiness was involved in genome-wide and/or locus-specific genetic divergence.

## Methods

### Ethics statements

*E*. *falcata* leaf samples were collected in two study sites of the Eastern Guiana shield: Laussat (5°28’37”N; -53°34’36”W) and Régina (4°18’44”N; -52°14’6”W). The study sites are managed by the French National Forests Office (ONF) which authorized tree labelling and leaf sampling. *E*. *falcata* is not a protected or endangered species and we certify that our experiment complies with the laws and ethical recommendations of France and French Guiana.

### Species description, study sites and sampling

**Fig 1:**
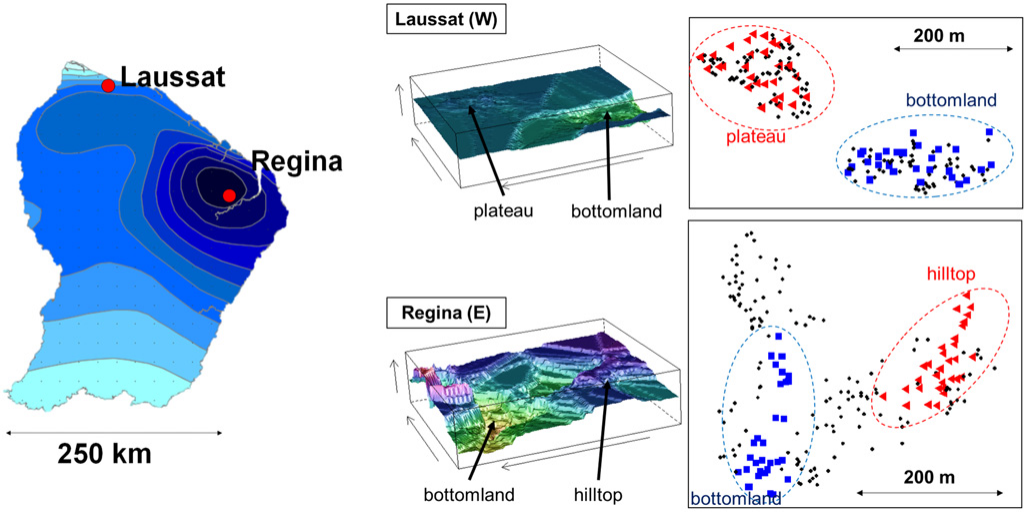
Geographic and topographic situation of the study sites. Colored dots: trees sampled for genotyping (triangles: hilltop; squares: bottomland).

**Fig 2:**
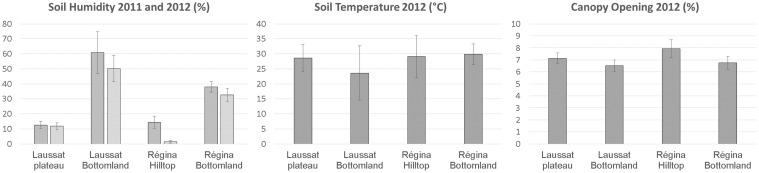
Environmental conditions in the study sites and local habitats: soil humidity (%), soil temperature (°C) and canopy opening (%). Complete data are provided in S1 Table.

*Eperua falcata* (Aublet) is a canopy-subdominant tree species, hyper-abundant in the Guiana shield [31]. Its distribution is aggregative, and aggregates often reach high population densities. Pollination is ensured by bats while seed dispersal is autochorous [32]: heavy seeds are dispersed at short distance around crowns of mother trees through explosive dehiscence. Our study includes two *E*. *falcata* populations located near the coast of the Eastern Guiana shield: Laussat (5°28’37”N; -53°34’36”W) and Régina (4°18’44”N; -52°14’6”W). These populations experience contrasted rainfall regimes, with a mean annual precipitation of 2500 mm and 3500 mm respectively (in years 2010 and 2011), and with a harsher dry season in Laussat (data from météo-FRANCE stations of ‘Iracoubo’ and ‘Régina’), Fig. 1. Both sites harbor different habitat types, from a bottomland to *terra firme*, and differ in landscape raggedness. In Laussat, a permanently water-logged bottomland gently rises toward a plateau of low elevation. In Régina, narrow seasonally flooded bottomlands and streambeds lie at the foot of hills and higher-elevation plateaus with steeper slopes. In both sites, bottomlands are characterized by hygromorphic soils with a large accumulation of organic matter up to a depth of 1 m caused by intense waterlogging, while *terra firme* are composed of well-drained ferralitic soils, rich in iron oxides with a sand-clay texture allowing free vertical drainage (S1 Fig.). Soil humidity and temperature were assessed at the end of the dry season (in 2011 and 2012) in each study site and local habitat using a soil moisture sensor TRIME-PICO32 (Table 1, Fig. 2 and S1 Table). Canopy opening was estimated by realizing fisheye hemispherical photographs with a Nikon digital camera and treated using Gap Light Analyzer V2.0 [33] to estimate canopy opening, Leaf Area Index (LAI) and the total light transmitted to ground (Fig. 2, S1 Fig., and S1 Table).

**Table 1:** Summary of the environmental conditions in the study sites and local habitats: soil type, waterlogging frequency and seasonal soil drought severity.

Site and local habitat	Laussat Plateau	Laussat Bottomland	Régina Hilltop	Régina Bottomland
Soil Type	Ferralitic	Hygromorphic	Ferralitic	Hygromorphic
Waterlogging frequency	no	permanent	no	seasonal
Seasonal Soil Drought Severity	strong	low	very strong	intermediate

Complete data are provided in S1 Table (see also Fig. 2).

All trees of diameter at breast height (d.b.h.) > 20 cm were mapped in a continuous area of 6.7 ha in Régina, and in two areas of 2.5 ha and 1.8 ha in Laussat. Population density varied between 29.9 adult trees/ha and 48.11 trees/ha in Régina and Laussat respectively. In each site, two groups of 30 trees inhabiting distinct habitat types (named ‘bottomland’ and ‘hilltop’, Fig. 1) were randomly selected and sampled for genetic analyses, totaling 120 trees with elevations ranging from 17 to 60 m a.s.l. in Laussat and from 47 to 92 m a.s.l in Régina. The sample size was set to 30 trees per population, in agreement with the intermediate sample size simulated by Foll and Gaggiotti [34] to test the powerful of their method to detect dominant loci under selection. Tree descriptions (site, local habitat, coordinates) are accessible on Dryad (http://dx.doi.org/10.5061/dryad.b2q88).

### Molecular methods

Genome-scans are very powerful for apprehending the extent of genome-wide genetic differentiation in wild populations and for detecting locus-specific signatures of population divergence [35] which can be interpreted as suggestive of the action of natural selection [36]. In non-model species, AFLP markers [37] are widely used for genome-wide analyses of within-population genetic variation [38–43]. Despite the drawbacks of being dominant and anonymous, they have been proved to outperform other markers-such as micro-satellites- in the detection of genetic structure [44] and in the discrimination of taxa and populations [45, 46]. They present the obvious advantage of being easily obtained, relatively robust, and of requiring no prior sequence information [45–48]. These markers have, however, been largely criticized for their lack of reproducibility [49] and require rigorous strategies to check repeatability and to control for genotyping errors [50].

Fresh leaves were sampled and frozen at -80°C as soon as they arrived to the lab (in the evening following the sampling). Genomic DNA was extracted using a CTAB protocol [51, 52], and each sample was extracted twice independently. Amplified fragment length polymorphisms (AFLPs) profiling was performed according to the protocol of Vos, Hogers and Bleeker [37]. DNA was digested using *Pst*I and *Mse*I restriction enzymes [37, 53–55]. Restriction fragments were amplified through two selective PCRs with respectively one and three selective nucleotides. Fifteen primer combinations were analyzed: *Pst*+ACA/*Mse*+TAA, *Pst*+ATT/*Mse*+TAA, *Pst*+AAC/*Mse*+TAA, *Pst*+ATA/*Mse*+TAA, *Pst*+ACA/*Mse*+TAG, *Pst*+ATT/*Mse*+TAG, *Pst*+AAC/*Mse*+TAG, *Pst*+ATA/*Mse*+TAG, *Pst*+TAA/*Mse*+CAA, *Pst*+TAG/*Mse*+CAA, *Pst*+ACA/*Mse*+CAA, *Pst*+ATA/*Mse*+CAT, *Pst*+ACA/*Mse*+CAT, *Pst*+ATT/*Mse*+CAT, *Pst*+ATA/*Mse*+CAT. The complete protocol (including DNA extraction, AFLP protocol and genotyping) was realized twice independently for each sample to obtain a complete replicate of the dataset (totaling 2 x 120 trees = 240 samples).

AFLPs were scored through an automated cleaning procedure (encoded in R): (i) negative controls were used to define thresholds of peak detection, (ii) peak profiles were scanned using PeakScanner Software v1.0 (Applied Biosystems) and the bin set was created using RawGeno v2.0 [47] with the previously defined thresholds, (iii) a consensus AFLP profile was edited for each sampled tree (only well replicated genotypes were kept, genotypes that were not replicated were considered as missing), (iv) data were post-cleaned, in particular by removing markers that were not genotyped in at least 15 trees per site/local habitat combination. The complete method of AFLP scoring is available in the S1 Method; AFLPs data (binary) are accessible on Dryad (http://dx.doi.org/10.5061/dryad.b2q88).

### Genetic structure and spatial genetic structure analysis (SGS)

A Bayesian clustering analysis was performed using STRUCTURE v2.3.4 [56] at both regional and local scale. The analyses were performed with the ‘admixture model’ and ‘correlated allelic frequencies’ settings. A burn-in of 10,000 iterations was followed by 100,000 iterations. As we had no *a priori* expectation about the number of clusters to be inferred, the model was run with *K* (number of clusters) values from *K* = 1 to *K* = 10 (five runs were performed for each *K* value). Trends in L(*K*) were analyzed using R software, in accordance with the ad-hoc *ΔK* method proposed by Evanno, Regnaut and Goudet [57]. STRUCTURE results were summarized using CLUMPAK server [58] to obtain the probability of each individual to belong to each cluster.

Spatial genetic structuring and gene dispersal were assessed on AFLP data using the spatial autocorrelation method based on kinship coefficients, as developed by Hardy and Vekemans [59] and implemented in SPAGeDi v1.3 [60]. Within each site, spatial autocorrelation of kinship coefficient (*F*_ij_) was analyzed over twenty evenly spaced distance classes between 0 and 500 m. 95% *null* confidence intervals were obtained through 1000 random permutations of individuals among geographical locations. Neighborhood size (*N*_b_) and gene dispersal (*σ*_g_) were estimated with prior knowledge about population densities, and the slope (*b*) of the regression of kinship relatedness (*F*_ij_) against geographic distance (*d*_ij_) was computed with standard error estimated by jack-knifing over loci. SGS intensity was measured as *S*_p_ = *b*/(*F*_(1)_-1) where *F*_(1)_ is the average kinship coefficient between individuals separated by distances belonging to the first distance class.

### Landscape-scale analysis of genome-wide divergence

A landscape approach was used to test whether environmental variations were involved in genome-wide genetic divergence. The simultaneous effects of neutral and adaptive sources of genetic divergence were explored through a linear model. More precisely, the model aimed at distinguishing the relative influence of geographic and environmental distances on genetic distance between individuals. Neutral components were estimated both at the regional (based on the membership of individuals relative to different sites) and local scales (based on individual coordinates in a two-dimensional x,y-plane and along a one-dimensional elevation gradient). Adaptive components were modelled through the environmental distance between individuals (soil type, waterlogging frequency and seasonal drought strength). Because light and soil temperature were poorly variable among sites and local habitats, these two factors were excluded from the model (Fig. 2 and S1 Table).

$${GENET}_{i1,i2}\;=\;\mu +\left(\theta_{1}\;\times \;{SITE}_{i1,i2}\right)+\left(1-{SITE}_{i1,i2}\right)\left[\left(\theta_{2}\times {GEO}_{i1,i2}\right)+\left(\theta_{3}\times {ELEV}_{i1,i2}\right)\right]+\left(\theta_{4}\times {DROUGHT}_{i1,i2}\right)+\left(\theta_{5}\times {WATERLOG}_{i1,i2}\right)+\left(\theta_{6}\times {SOIL}_{i1,i2}\right)+\sigma_{R}^{2}$$

Where GENET_i1,i2_ is the genetic distance between the individuals i1 and i2 (Jaccard distance), μ is the global mean, and σ²_R_ the residual variance. SITE _i1,i2_ describes whether the individuals i1 and i2 are from the same site or not (0 = same site, 1 = different sites), GEO_i1,i2_ and ELEV_i1,i2_ are the geographic (Euclidean) distances between individuals inhabiting the same site according to their two-dimensional coordinates in the x,y-plane and their one-dimensional coordinates along an elevation gradient respectively. DROUGHT_i1,i2_, WATERLOG_i1,i2_, and SOILTYPE_i1,i2_ describe the environmental distances between individuals for seasonal drought severity, waterlogging frequency and soil type, according to sites and local habitat environmental properties as described in Table 1. The model was empirically calibrated through a Bayesian method implemented in OpenBUGS [61, 62] (http://www.openbugs.net): 10,000 iterations with a burning of 1,000. A complete description of the model and the BUGS code are provided in S2 Method.

### Allele frequency inference

Because properly estimating genotypic frequencies from dominant markers requires prior knowledge of inbreeding coefficient, *F*_IS_ was estimated from an already published dataset composed of SNPs detected in sequenced ESTs [8] with ARLEQUIN v3.5.1.2 [63]. The mean *F*_IS_ (across loci) varied from -0.207 to -0.089 depending on the population considered. Allele frequencies within each study site and local habitat were inferred from AFLPs data based on a mean inbreeding coefficient of -0.14, by solving the standard equation relating inbreeding coefficient, allele frequencies and recessive genotype frequencies for each marker *j*, with *f*(00) the relative frequency of the genotype (00) and *p* the relative frequency of the ‘0’ allele:

$$f{\left(00\right)}_{j}\;=\;\left(1-F_{IS}\right)p_{j}^{2}+\left(F_{IS}p_{j}\right),$$

Solving for p:

$$p_{j}\;=\;\frac{-F_{IS}+\sqrt{\Delta_{j}}}{2(1-F_{IS})}$$

with

$$\Delta_{j}\;=\;F_{IS}²-[4\left(1-F_{IS}\right)\left(-{f\left(00\right)}_{j}\right)]$$

Absolute frequencies were obtained by multiplying relative frequencies by twice the sample size in each subpopulation, rounding to the nearest integer. These absolute frequencies were used in all subsequent analyses of population differentiation and outlier detection.

### Intra-site differentiation

For each study site, locus-specific genetic differentiation (*F*_ST_) between local habitats was estimated from inferred genotypic data through a classical analysis of molecular variance (AMOVA [64]) using ARLEQUIN v3.5.1.2 (Slatkin’s method).

### Detection of outlier loci

Excess divergence within populations inhabiting contrasting habitats was tested based on two *F*_ST_-based approaches:

- the coalescent-based FDIST method [65] implemented in ARLEQUIN v3.5.1.2 [63]. We implemented both a hierarchical island model including the two study sites simultaneously, plus two classical island models for each site separately (within-Laussat and within-Régina respectively). False-discovery rate was assessed according to Strimmer’s method [66, 67]: p-values obtained from the coalescent method were converted into q-values using the ‘*fdrtool*’ package in R [67], and the latter was used to set an FDR threshold of 0.10.
- the Bayesian method implemented in BAYESCAN [34], with an FDR threshold of 0.10.

For each outlier detected, X² tests were performed on AFLP band frequencies to test the hypothesis of equal frequencies between local habitats within each study site.

### Evaluating Type I and Type II error rates

Both the Bayesian- and the coalescent-based methods were submitted to a sensitivity analysis by estimating Type I and Type II error rates. To do this, we simulated one-hundred datasets with the same sample size and number of markers as our empirical datasets (two groups of two populations with divergence between groups *F*_CT_ = 0.01). Out of the 1196 simulated markers, 1146 were simulated with average *F*_ST_ = 0.039 and *F*_ST_ = 0.026 (equal to empirical within-site *F*_ST_ values, corresponding to α = 0 in the Bayesian framework), 25 were constrained at *F*_ST_ = 0.11 (Bayesian α = 3) and 25 at *F*_ST_ = 0.23 (Bayesian α = 5) to simulate zero, moderate and strong selection respectively. The simulations were submitted to the same outlier detection analyses as the empirical dataset, and the average number of significant markers in each class, over the global set of one-hundred simulations, were reported. The ratio of number of neutral markers detected as significant, over the total number of neutral markers, was taken as an estimate of Type I error rate. The number of markers under selection not detected as significant, out of the total number of markers under selection, was taken as an estimate of Type II error rate.

## Results

### AFLP data

After data cleaning, 53.3% of markers (corresponding to 1196 bins out of 2242) were retained for further analysis as described in S1 Method. The binset is available on Dryad (http://dx.doi.org/10.5061/dryad.b2q88).

### Blind analysis of population structure

L(*K*) was high from *K* = 1 to *K* = 7 for the regional-scale analysis (S2 Fig.), from *K* = 1to *K* = 7 within Laussat, and from *K* = 1to *K* = 5 within Regina (S3 and S4 Figs.). At the regional level, a maximum peak of *Δ*K was detected at *K* = 3: individuals from ‘Régina’ were assigned to one cluster, while the individuals from ‘Laussat’ were assigned to two clusters concordant with local habitats (S2 Fig.). At *K* = 2, the inferred clusters distinguished the trees inhabiting the two study sites of Laussat and Regina. At intra-site level, a maximum peak of *ΔK* was detected at *K* = 2 in Laussat, and at *K* = 5 in Régina (S3 and S4 Figs.). In Laussat, the genetic clusters inferred at *K* = 2 were geographically grouped in agreement with local habitat patchiness (S3 Fig.), except for five trees of hilltop assigned to the same genetic clusters than the trees inhabiting the bottomland. In Régina the genetic structure was not clear at *K* = 5 as the individuals were assigned to the different clusters with quasi-equal probabilities, indicating a complete admixture and the probable absence of genetic structuring (S4 Fig.).

### Spatial Genetic Structure and gene dispersal within populations

**Fig 3:**
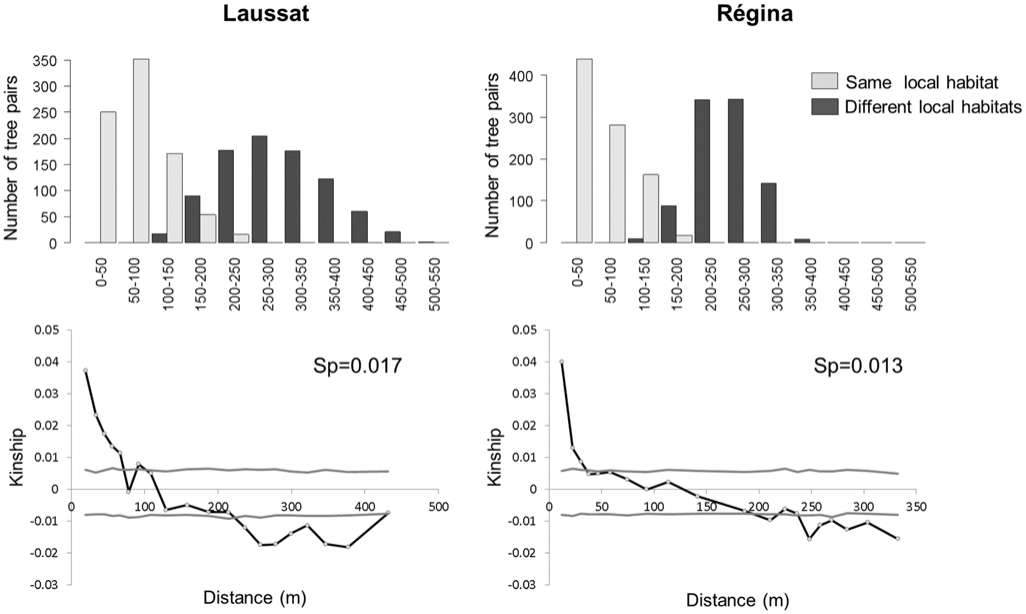
Top: Number of tree pairs in each distance class. Bottom: Intra-site spatial genetic structure (SGS) analysis based on all AFLP markers.

Spatial genetic structure (SGS) was assessed by estimating relative relatedness in 1711 pairs of individuals in Régina and 1810 pairs in Laussat. The mean number of pairs by distance class was 86 in Laussat and 92 in Régina. Significant SGS were detected in both sites (Fig. 3 and Table 2), with kinship declining with increasing geographical distance (b = -0.016 ±0.001 in Laussat and b = -0.012 ±0.001 in Regina). In Laussat, spatial autocorrelation was significantly positive until 56 m, and became significantly negative from 230 m onward, with a neighborhood size (*N*_b_) of 65.6. In Régina, autocorrelation was positive and significant until 30 m and became negative and significant beyond 250 m, with a neighborhood size *N*_b_ = 78.5. In both sites, the autocorrelation was positive at a distance corresponding to the distance separating trees inhabiting the same habitat, and became negative at a distance corresponding to the distance separating trees inhabiting two distinct habitats. Gene dispersal was estimated at 45.7 and 64.4 m in Laussat and Régina respectively. We also checked that spatial genetic structure did not vary among local habitats: significant SGS were detected in both habitats until 20 and 30 m in Laussat, and until 20 m in Régina (S5 Fig.), and we did not detect differences in SGS among local habitat types within sites, on the basis of the extent of relatedness (*F*_i,j_), SGS intensity (*S*_*p*_) and slope (b), S5 Fig.

**Table 2:** SGS and gene dispersal parameters estimated by SpaGeDi.

	Parameter	Laussat	Regina
**SGS parameter estimates**	b (SE)	-0.016 (0.001)	-0.012 (0.001)
	*F1* (SE)	0.037 (0.003)	0.04 (0.032)
	*Sp*	0.017	0.013
**Gene dispersal parameter estimates**	D	0.005	0.003
	Nb (SE)	65.62 (21.02)	78.51 (14.21)
	σg (SE)	45.7 (7.33)	64.4 (5.82)

*F*1 is the autocorrelation of kinship coefficient in the first distance class, b is the slope of the regression between relatedness (*F*_*ij*_) and geographic distance (*d*_*ij*_), *Sp* is SGS intensity, D is population density, Nb is Neighborhood size, and σg is gene flow estimate.

### Landscape scale analysis of genetic divergence

**Fig 4:**
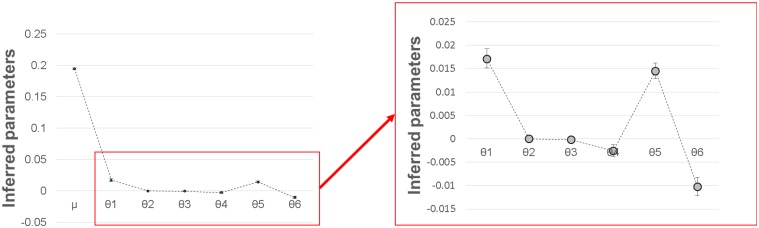
Results of the landscape-scale Bayesian model (values are provided in Table 3). Points show the inferred parameters with their 95% posterior probability: μ is the global mean, θ _1_ represents the effect of site, θ_2_ is the slope of the relation between the geographical and genetic distance within sites in a two-dimensional x,y-plane, θ_3_ is the slope of the relation between the geographical and genetic distance within sites according to an elevation gradient, θ_4_ describes the effect of drought severity, θ_5_ describes the effect of waterlogging frequency and θ_6_ describes the effect of soil type.

Partitioning the genetic distance into neutral and adaptive processes through a landscape Bayesian model revealed a strong ‘site’ effect on the genetic distance between individuals (θ1 = 1.71.10^-2^, Table 3 and Fig. 4). Within site, we detected a positive relationship between the geographic distance in the two-dimensional x,y-plane and the genetic distance between individuals: θ2 = 4.2.10^-5^ m^-1^ (i.e. the mean genetic distance between individuals increases of 0.042 every kilometer). However, there was no positive trend elevation and genetic distances. Among environmental sources of genetic divergence, waterlogging frequency was positively related with genetic distance (θ5 = 1.5.10^-2^).

**Table 3:** Parameters inferred by the landscape Bayesian model with their respective posterior probabilities (mean, standard deviation, median, and 95% credible interval): μ (global mean), θ_1_ (site effect), θ_2_ (slope of the relation between geographical and genetic distance within sites according to a 2D x,y-plane), θ_3_ (slope of the relation between the geographical and genetic distance within sites according to an elevation gradient), θ_4_ (drought severity effect), θ_5_ (waterlogging frequency effect), and θ_6_ (soil type effect).

	mean	sd	val2.5pc	median	val97.5pc
μ	1.95×10^-01^	6.82×10^-04^	1.93×10^-01^	1.95×10^-01^	1.96×10^-01^
θ1	1.70×10^-02^	1.03 ×10^-03^	1.52×10^-02^	1.71×10^-02^	1.92×10^-02^
θ2	4.19×10^-05^	5.81×10^-06^	3.05×10^-05^	4.19×10^-05^	5.32×10^-05^
θ3	-1.85×10^-04^	5.86×10^-05^	-2.98×10^-04^	-1.85×10^-04^	-6.93×10^-05^
θ4	-2.57×10^-03^	6.59×10^-04^	-3.88×10^-03^	-2.56×10^-03^	-1.29×10^-03^
θ5	1.45×10^-02^	8.54×10^-04^	1.28×10^-02^	1.45×10^-02^	1.62×10^-02^
θ6	-1.02×10^-02^	9.91×10^-04^	-1.22×10^-02^	-1.02×10^-02^	-8.23×10^-02^

### Genetic differentiation among subpopulations inhabiting contrasted habitats and outlier detection

**Fig 5:**
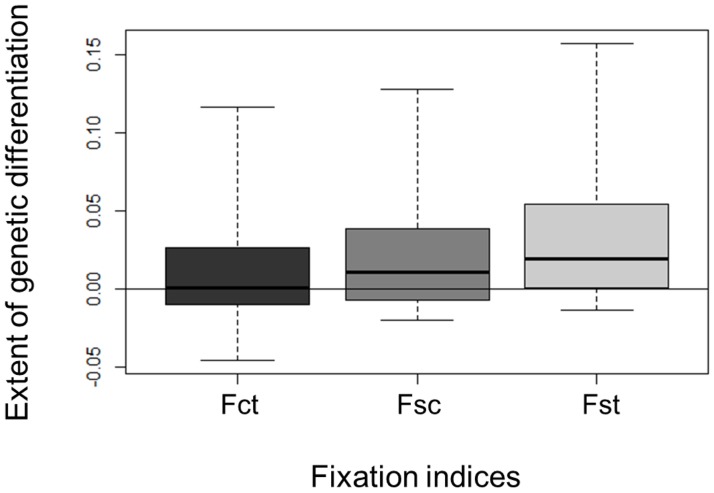
Box-plot of the distribution of single-locus fixation indices (*F*_*CT*_, *F*_*SC*_ and *F*_*ST*_) estimated under the hierarchical model of population subdivision (boxes indicate 5%, 25%, 50%, 75% and 95% quantiles). *F*_*CT*_ is the differentiation between sites relative to total, *F*_*SC*_ is the differentiation between local habitats relative to sites, and *F*_*ST*_ is the differentiation between local habitats relative to total.

Overall Slatkin’s *F*_ST_ between local habitats was respectively 0.03 (sd = 0.07) in Laussat and 0.02 (sd = 0.05) in Régina (S6 Fig.). Locus-specific *F*_*ST*_ was significant for 8.1% and 6.1% of loci in Laussat and Régina respectively. Under the hierarchical island model, the extent of differentiation between study sites was *F*_CT_ = 0.01 (sd = 0.05), while the differentiation between local habitats within sites was *F*_SC_ = 0.03 (sd = 0.05) and the differentiation between local habitats among sites *F*_ST_ = 0.04 (sd = 0.06), Fig. 5.

**Fig 6:**
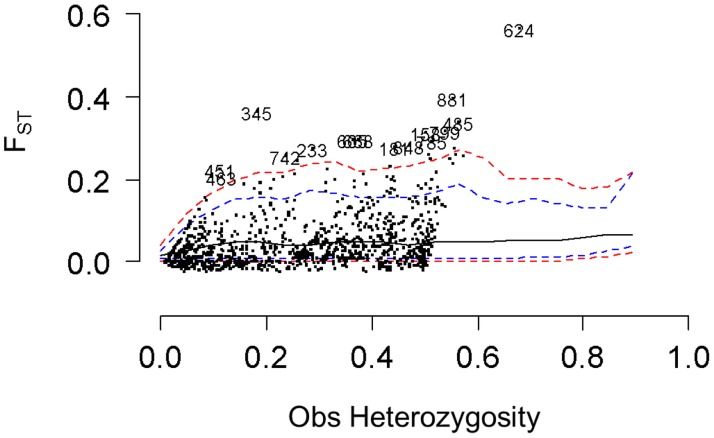
Results of the coalescent outlier search under the hierarchical island model. Blue dashed line: 95% neutral envelop; red dashed line: 99% neutral envelop. Only loci above the neutral envelop and retained after multiple corrections (FDR = 10%) are shown.

**Fig 7:**
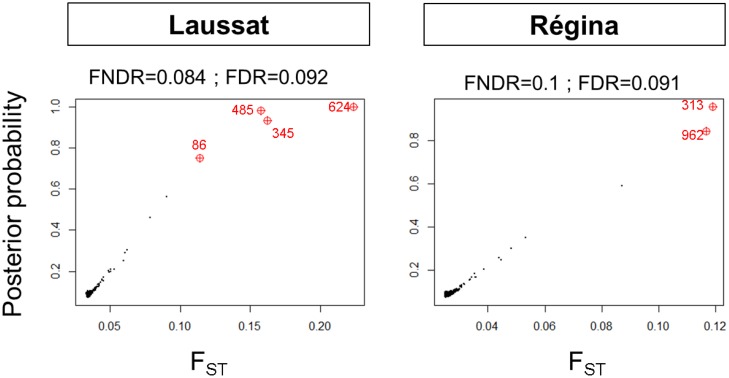
Results of the Bayesian outlier search under a 10% expected FRD.

After local false-discovery rate assessment [66, 67], 42 loci were detected as outliers being under divergent selection in at least one analysis (under a FDR threshold of 10%), Table 4. Under the hierarchical coalescent model, fifteen (1.25%) outlier loci were detected between subpopulations within regions (F_ST_), Fig. 6 and Table 4 (column 2). The within-site coalescent analyses revealed fifteen (1.35%) and eighteen (1.65%) outliers respectively for Laussat and Régina (out of 1109 and 1090 polymorphic markers respectively), Table 4 (columns 6 and 11). Among all outliers detected by the hierarchical model, four were also detected by the within-site coalescent model in Laussat (loci 345, 485, 624 and 742), and one in Régina (locus 463). Locus 46 was detected by both within-site analyses but not in the hierarchical model. The Bayesian analysis detected four outliers (loci 86, 345, 485 and 624, FDR = 0.084 and FNDR = 0.092) in Laussat, and two (loci 313 and 962, FDR = 0.01 and FNDR = 0.091) in Regina (Fig. 7 and Table 4, columns 7 and 12). All outliers detected by the Bayesian methods were also detected by within-site coalescent analyses (loci 86, 345, 485 and 624 in Laussat, loci 313 and 962 in Régina), and three (loci 345, 485 and 624) by the hierarchical model as well. Simulations were used to assess Type I and Type II error rates. For the coalescent method, Type I error rate was α = 0.3% and Type II error rate was β = 57% for both sites; error rates were similar for the Bayesian method (α = 0.3% and α = 0.2% for Laussat and Régina respectively; β = 54% for both sites).

**Table 4:** Summary of outliers detected in at least one analysis.

Locus	Hierarchical approach Coalescent outlier detection under a hierarchical model (1196 polymorphic markers)	Within-Laussat classical approach (1109 polymorphic markers)					Within-Regina classical approach (1090 polymorphic markers)				
		Slatkin FST within Laussat	Fst Pval	X² test on AFLP band frequency within Laussat (p-value)	Coalescent outlier detection under a classical island model within Laussat	Bayesian outlier detection within Laussat	Slatkin FST within Regina	Fst Pval	X² test on AFLP band frequency within Régina (p-value)	Coalescent outlier detection under a classical island model within Régina	Bayesian outlier detection within Régina
19	ns	-0.014	0.454	0.6797	ns	ns	0.238	0.002	0.1094	*	ns
30	ns	0.184	0.001	0.1084	ns	ns	0.229	0.001	0.09	*	ns
41	ns	-0.029	1.000	1	Ns	ns	0.177	0.009	0.005*	*	ns
46	ns	0.290	0.000	0.036*	*	ns	0.311	0.000	0.0295*	*	ns
54	ns	0.148	0.011	0.072	ns	ns	0.336	0.000	0.0025*	*	ns
69	ns	-0.021	1.000	Na	ns	ns	0.304	0.000	0.1014	*	ns
86	ns	0.435	0.000	0.002*	*	*	-0.032	1.000	1	ns	ns
158	*	-0.020	0.801	0.7026	ns	ns	-0.017	0.718	0.7156	ns	ns
181	*	0.078	0.025	0.054	ns	ns	0.014	0.268	0.2649	ns	ns
221	ns	0.000	0.443	0.4123	ns	ns	0.339	0.000	0.009*	*	ns
233	*	0.002	0.458	0.4873	ns	ns	0.090	0.019	0.0395*	ns	ns
288	ns	0.268	0.000	0.045*	*	ns	0.046	0.160	1	ns	ns
291	ns	-0.024	1.000	1	ns	ns	0.218	0.000	0.0375*	*	ns
313	ns	0.042	0.114	0.2854	ns	ns	0.417	0.000	0.0005*	*	*
345	*	0.383	0.000	0.0005*	*	*	na	na	na	na	na
359	ns	-0.005	0.430	0.5557	ns	ns	0.231	0.000	0.0005*	*	ns
416	ns	0.014	0.187	0.6192	ns	ns	0.222	0.001	0.0915	*	ns
451	*	0.200	0.000	0.002*	ns	ns	na	na	na	na	na
457	ns	0.350	0.000	0.006*	*	ns	-0.007	0.478	0.5797	ns	ns
463	*	na	na	na	na	na	0.265	0.000	0.0005*	*	ns
468	ns	0.334	0.000	0.0115*	*	ns	0.135	0.006	0.0595	ns	ns
485	*	0.548	0.000	0.0005*	*	*	-0.024	0.771	0.7251	ns	ns
585	ns	0.334	0.000	0.011*	*	ns	0.114	0.009	0.4838	ns	ns
605	*	0.017	0.500	0.4848	ns	ns	-0.018	0.833	1	ns	ns
624	*	0.638	0.000	0.0005*	*	*	0.129	0.010	0.2009	ns	ns
668	*	0.006	0.295	0.2779	ns	ns	-0.014	1.000	1	ns	ns
715	ns	-0.024	0.811	1	ns	ns	0.235	0.001	0.0895	*	ns
742	*	0.315	0.000	0.0005*	*	ns	-0.018	1.000	1	ns	ns
743	ns	0.329	0.001	0.001*	*	ns	-0.031	1.000	1	ns	ns
748	ns	0.129	0.005	0.0865*	ns	ns	0.290	0.000	0.009*	*	ns
757	ns	0.307	0.000	0.001*	*	ns	0.089	0.033	0.0465*	ns	ns
785	*	0.239	0.001	0.005*	ns	ns	0.066	0.096	0.1434	ns	ns
791	ns	0.035	0.100	0.3653	ns	ns	0.294	0.000	0.035*	*	ns
799	*	-0.023	1.000	na	ns	ns	0.002	0.311	0.4253	ns	ns
848	*	0.195	0.002	0.0405*	ns	ns	-0.022	1.000	na	ns	ns
860	ns	0.388	0.000	0.0005*	*	ns	0.095	0.009	0.0425*	ns	ns
868	ns	0.354	0.000	0.006*	*	ns	0.020	0.236	0.4113	ns	ns
871	ns	0.209	0.000	0.001*	ns	ns	0.255	0.000	0.0005*	*	ns
874	ns	-0.016	1.000	1	ns	ns	0.223	0.000	0.0005*	*	ns
881	*	0.226	0.001	0.007*	ns	ns	0.002	0.319	0.3113	ns	ns
955	ns	0.301	0.002	0.0355*	*	ns	0.006	0.334	0.3533	ns	ns
962	ns	-0.017	1.000	1	ns	ns	0.257	0.000	0.0005*	*	*

*: significant

ns: non-significant

na: missing value

**Fig 8:**
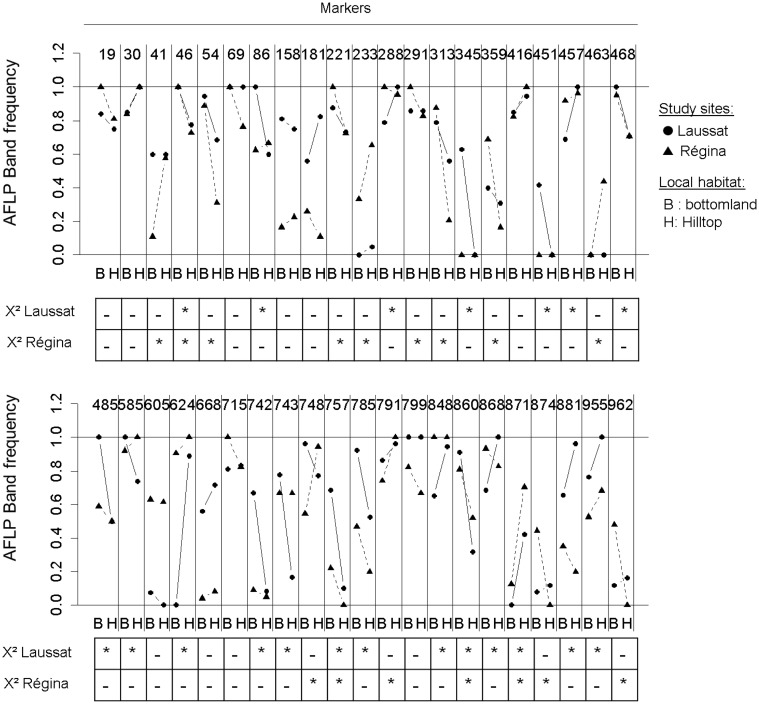
Band presence frequency (allele ‘1’) in each local habitats (‘B’: bottomland, ‘H’: hilltop) and each study site (circles: Laussat, squares: Régina). The tables below show the result of X² tests on AFLP band frequency ('*': significant; ‘-’: non-significant or missing).

To check whether variations in AFLP band frequencies between local habitats were consistent with the hypothesis of selection acting in the same direction in the two replicates, we compared the direction of inter-habitat variation in band frequencies between the two study sites for outlier loci, Fig. 8. About half of all detected outliers showed the same trend of frequency variations in the two study sites. For loci 30, 233, 416, 624, 668, 785, 791, 871 and 955, the frequency of ‘1’ (band presence) was higher in hilltop than in bottomland in both sites. For loci 19, 46, 54, 221, 313, 359,468, 485, 757, and 860, the frequency of ‘1’ was higher in bottomland than in hilltop in both sites. However, X² tests revealed significant differences in AFLP band frequency between local habitats in at least one study site for only fifteen outliers (46, 54, 221, 233, 313, 359, 468, 485, 624, 757, 787, 791, 860, 871 and 955). Finally, congruent patterns of AFLP band divergence between local habitats were supported by significant X² tests in the two study sites for only three outliers: 46, 757 and 871. Six loci were monomorphic in one study site: loci 69, 345, 451, 463, 799 and 848.

## Discussion

The genetic clusters inferred by STRUCTURE were spatially aggregated. At regional scale, the genetic structuring among populations inhabiting different study sites (*K* = 2) can easily be explained by isolation-by-distance. To evaluate the role of neutral processes in shaping within-population genetic structure, we investigated the fine-scale genetic structuring over all loci within each study site. Kinship coefficients decreased with geographical distances in the two study sites as expected under the isolation-by-distance model. Gene flow estimates were very low in both sites (around 50 m), and the lower gene flow estimated in Laussat (45.7 m against 64.4 m in Régina) was concordant with the stronger genome-wide genetic structuring among local habitats in that site. Similar SGS patterns have been observed in many temperate [14, 39, 42, 68–72] and tropical tree species [73–79] (including the Guiana shield [13, 20, 80–82]), and they are likely to be caused by neutral processes (restrictions in gene flow and local inbreeding [80]). In tropical trees, pollen dispersal is commonly restricted to short distances, and seed dispersal is often highly restricted in autochorous species [13, 16, 22] causing the clumping of maternal progeny groups. Consequently, mating among neighboring relatives—which may be frequent in dense populations—commonly results in local inbreeding. In autochorous *E*. *falcata*, gene dispersal estimates (σ_g_ ranging from 45.7 to 64.4 m depending on the study site) are in agreement with these characteristics [71, 83].

The slight variations in the fine-scale spatial genetic structuring between study sites and between local habitats within sites may possibly be caused by variations in environmental conditions and their direct effect on pollen and seed dispersal. For example, fine-scale spatial genetic structure and population differentiation were weaker, and gene flow was slightly higher, in the study site of Régina (where the relief is steep with abrupt slopes and precipitation more abundant with about 3500 mm/year) than in Laussat (where the relief is quite flat and precipitation does not exceed 2500 mm/year). Variations in topography (relief and slopes), rainfall and water flows may have a direct effect on gene flow and SGS. Even if the spatial genetic structuring was quite similar among habitat types, it was slightly weaker in the bottomland than in the plateau in Laussat, possibly because water flows caused by intense waterlogging contribute to scatter seeds and to increase gene flow in this habitat.

The existence of genome-wide neutral divergence directly caused by geographic distances (between and within study sites) was also corroborated by the landscape-scale analysis of genetic divergence. Indeed, the inferred parameter θ1 revealed a site effect on the genetic divergence. Even if it is more likely to be caused by neutral processes related to the geographic distance itself (isolation-by-distance), this parameter may also capture genome-wide adaptive processes caused by variations in both abiotic and biotic conditions between the study sites (e.g. variation in rainfall, population density, competition levels, etc.). The parameter θ2 captured the effect of geographic distances on genetic distances within study sites (in the two-dimensional x,y-plane), confirming the existence of a neutral spatial genetic structuring within study sites.

In addition to neutral, distance-based, genome-wide divergence, variations in waterlogging frequency may constitute a source of adaptive genetic divergence as revealed by a positive estimate of the parameter θ5. Surprisingly, the waterlogging effect was of the same order of magnitude as the site effect, despite the large differences in geographical scales separating sites (hundreds of kilometers) and local habitats (hundreds of meters). This implies that a fraction of genome-wide divergence may have been caused by ‘pervasive selection’ [8, 69] over microgeograhical scales, as expected under the isolation-by-adaptation (IBA) model [84]. Indeed, indirect estimates of gene flow in well-established adult populations represent the ‘effective’ gene flow. They do not depend on seed and pollen dispersal only, but also on the ability of seedlings to establish and grow in the environment where they were dispersed (i.e. on local adaptation processes), that is particularly true for immobile organisms. The genetic differentiation between local subpopulations (*F*_ST_ = 0.04; sd = 0.06) was large regarding the differentiation between sites (*F*_CT_ = 0.01; sd = 0.05), despite the geographical scales involved (about 300 km among sites, up to 200 m between local habitats). As the effects of dispersal limitation can only increase with distance, it seems unlikely that this kind of process would be stronger locally than at the regional level. This means that genome-wide divergence may be influenced by local adaptation to micro-environmental variability. In particular, waterlogging frequency influence genome-wide divergence over microgeographical scales as revealed by the landscape-scale approach, probably through its direct effect on seedlings establishment.

Locus-specific footprints of local adaptation were also detected for a fraction of the analyzed loci. Indeed, adaptive divergence may either affect many genes of low effects, or a reduced number of targeted loci (few genes of major effects) involved in key metabolic or physiologic pathways, themselves involved in fitness [85–92]. Both the coalescent and the Bayesian method allowed the detection of outliers at the microgeographical scale. Among the 42 (3.5%) outliers detected with the coalescent method, 6 were validated by the Bayesian method (0.5%, loci 85, 313, 345, 485, 624, 962) and are strong candidate targets of divergent selection [34, 65, 93]. Precision tests performed through simulations showed that false-positive outliers should be very rare (a fraction of a percent) making it unlikely that a large fraction of the detected outliers are artifacts. Nineteen interesting outliers showed similar trends of band frequency variations between local habitats in the two study sites: twelve were supported by significant variations in AFLPs band frequency among local habitats (X² test) in one study site, and three were supported by significant variations in AFLPs band frequency among local habitats in the two study sites. This result indicates that these outliers may be true positives and that divergent selection would have driven variations in genotypic frequencies among local habitats in the same direction in the two study sites. The majority of outliers were, however, detected in only one study site. This can be ascribe to a lack of statistical power and/or to environmental differences between the study sites. Indeed, simulations revealed that 50% of loci undergoing moderate to strong selection would go undetected. Alternatively, different selective pressures caused by different selective agents may be involved in the adaptive genetic divergence within the two study sites. Moreover, even assuming that the same selective agents occur in the two sites, different multi-locus combinations of alleles or different loci may have been selected in the two populations. In the case of traits under multi-genic control, it is hard to detect single targets of selection of low strength and to identify conserved single-locus divergence patterns [94].

Outliers may also indicate the presence of some other indirect mechanisms inducing genetic divergence that may not be directly related to environmental filters [65, 95, 96]. Outlier tests based on a differentiation index (*F*_ST_) are robust to inter-locus variations, and theoretical models show that footprints of natural selection persist longer in differentiation indices (*F*_ST_) than in intra-population estimators of genetic diversity [36]. *F*_ST_-based methods are also supposed to be robust to many demographic scenarios [97, 98], partly because demographic events affect the genome in a homogeneous manner [89]. However, the inclusion of bottlenecked populations may bias the method [36]. Even if trees were sampled in mature and supposedly undisturbed forests, we have no evidence that the studied populations have not experienced a recent demographic change (bottleneck or expansion), and the degree to which these tests are robust to demography has not yet been fully explored [99].

Scans for outlier detection are abundant in the literature for a variety of geographical scales and biological models, including animals and plants, both aquatic and terrestrial [8, 84, 91, 97, 100–107]. The proportion of outliers for selection detected was low, but surprisingly high when considering the microgeographical scale studied here. This suggests that the same processes that occur with a larger degree of spatial separation in other species may occur at very short distances in *E*. *falcata*. These loci may be involved in metabolic pathways crucial for seedlings establishment and growth under the particular constraints imposed by each habitat, such as waterlogging and hypoxia experienced in bottomlands, or seasonal soil drought experienced in plateaus. However, the selective agency behind the observed divergence needs to be functionally proven by showing that the putatively selected polymorphisms control adaptive traits. This will require (i) identifying the genes involved in fitness-related phenotypic traits, and (ii) targeting these loci for testing local adaptation on candidate genes [108].

Nevertheless, the patterns of divergence observed in this study are in agreement with previous reports based on SNPs within ESTs [8] and quantitative phenotypic traits [30], and reinforce the idea that adaptive phenomena may affect a substantial fraction of the genome at microgeographical scales in Neotropical tree populations. The example provided by *E*. *falcata* is a piece of evidence that evolution may drive genetic differentiation and subpopulation divergence even in conditions in which gene flow may easily erase the effects of weak selective forces (i.e. over microgeographical scales in continuous stands of high population densities with extensive gene flow). At such spatial scales, dispersal and population connectivity (which are the field of landscape genetics [109]) meet evolutionary processes (which are the field of population and ecological genetics) providing a deeper understanding of the ecological processes responsible for the maintenance of biodiversity. Indeed, adaptive divergence caused by microgeographic habitat patchiness may constitute the fuel that feeds the great diversity harbored by the tropical rainforest of Amazonia. The genetic diversity of wild populations in turn conditions their adaptive potential (i.e. their ability to adapt to environmental variations) and consequently their ability to persist when undergoing environmental changes. The present study suggests that understanding evolutionary processes in tropical rainforests and in plant populations more widely should require particular attention on microgeographic divergence and local adaptation.

## Supporting Information

S1 Fig Environmental characterization: Canopy opening and pedology. A: Example of hemispherical photograph done in the plateau of Laussat, B and C: Examples of soil toposequences (B: hygromorphic soil of Laussat bottomland, C: ferralitic soil of Laussat plateau).
(TIF)

S2 Fig Bayesian clustering analysis on the whole data set. Upper pane: L(*K*) and Δ*K* values. Middle pane: individual α values for *K* = 2 and *K* = 3. Lower pane: geographical distribution of individuals belonging to the main clusters (see text).
(TIF)

S3 Fig Bayesian clustering analysis on the Laussat data set. Upper pane: L(*K*) and Δ*K* values. Middle pane: individual α values for *K* = 2. Lower pane: geographical distribution of individuals belonging to the main clusters (see text).
(TIF)

S4 Fig Bayesian clustering analysis on the Régina data set. Upper pane: L(*K*) and Δ*K* values. Middle pane: individual α values for *K* = 5. Lower pane: geographical distribution of individuals belonging to the main clusters (see text).
(TIF)

S5 Fig Intra-habitat spatial genetic structure analysis based on all AFLP markers. (TIF)

S6 Fig Density distribution of Slatkin’s locus-specific *F*_ST_ overall loci and for loci displaying a significant F_ST_. (TIF)

S1 Method AFLP scoring. (DOCX)

S2 Method Model description and BUGS Code. (DOCX)

S1 Table Environmental conditions in each of the study sites and local habitat. (DOCX)
