# Correction: Neutral and Adaptive Drivers of Microgeographic Genetic Divergence within Continuous Populations: The Case of the Neotropical Tree *Eperua falcata* (Aubl.)

**DOI:** 10.1371/journal.pone.0127134

**Published:** 2015-04-22

**Authors:** The PLOS ONE Staff

## Notice of Republication

This article was republished on April 10, 2015, to correct Figs. 5, which were distorted during the production process. The publisher apologizes for the error. Please download this article again to view the correct version. The originally published, uncorrected article and the republished, corrected article are provided here for reference.

## Supporting Information

S1 File Originally published, uncorrected article. (PDF)

S2 File Republished, corrected article. (PDF)
