# Supplementary figures and images for: Neutral and Adaptive Drivers of Microgeographic Genetic Divergence within Continuous Populations: The Case of the Neotropical Tree Eperua falcata (Aubl.)
Source: PLoS One. 2015 Mar 25;10(3):e0121394. doi: 10.1371/journal.pone.0121394 (PMC4373894; doi:10.1371/journal.pone.0121394)

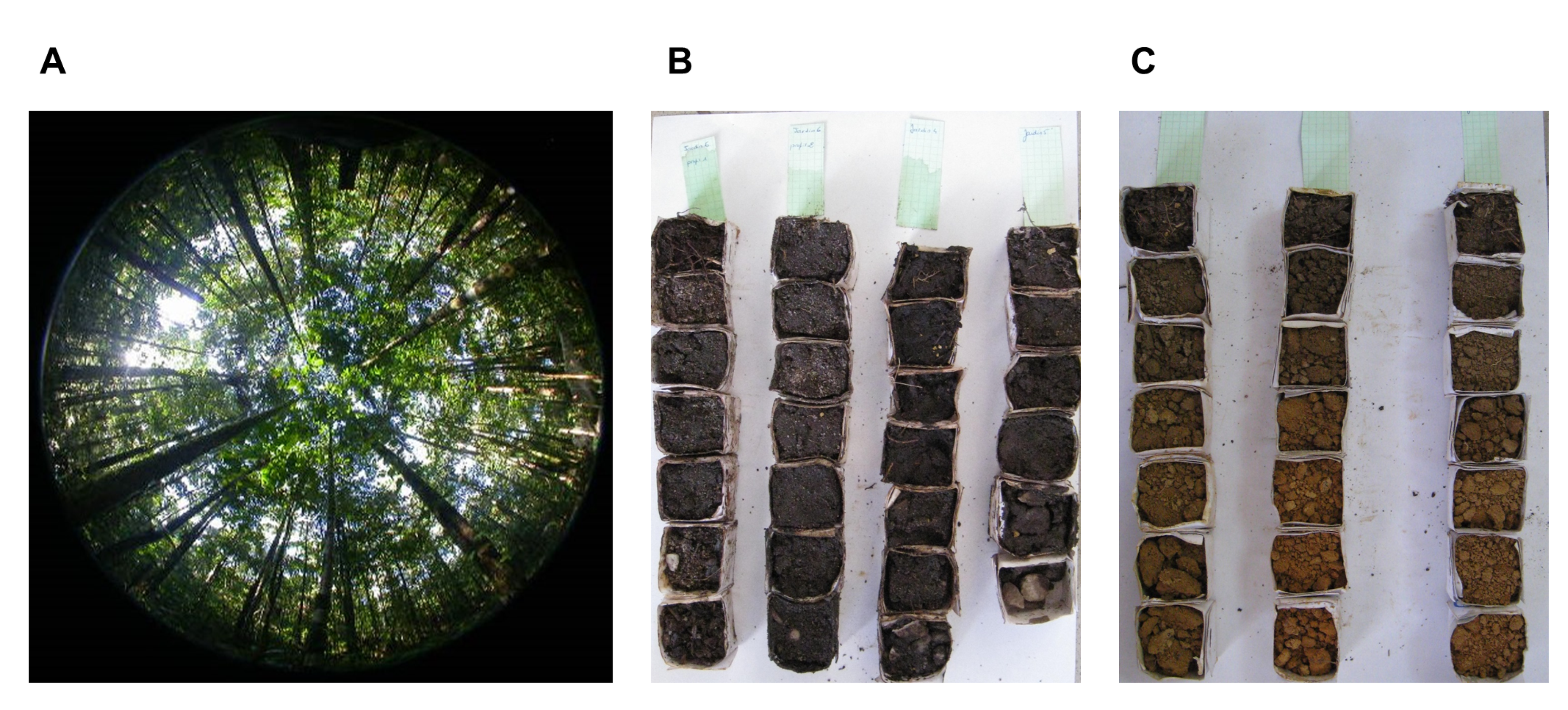

Supplement: S1 Fig — A: Example of hemispherical photograph done in the plateau of Laussat, B and C: Examples of soil toposequences (B: hygromorphic soil of Laussat bottomland, C: ferralitic soil of Laussat plateau). (TIF) [file pone.0121394.s001.tif]

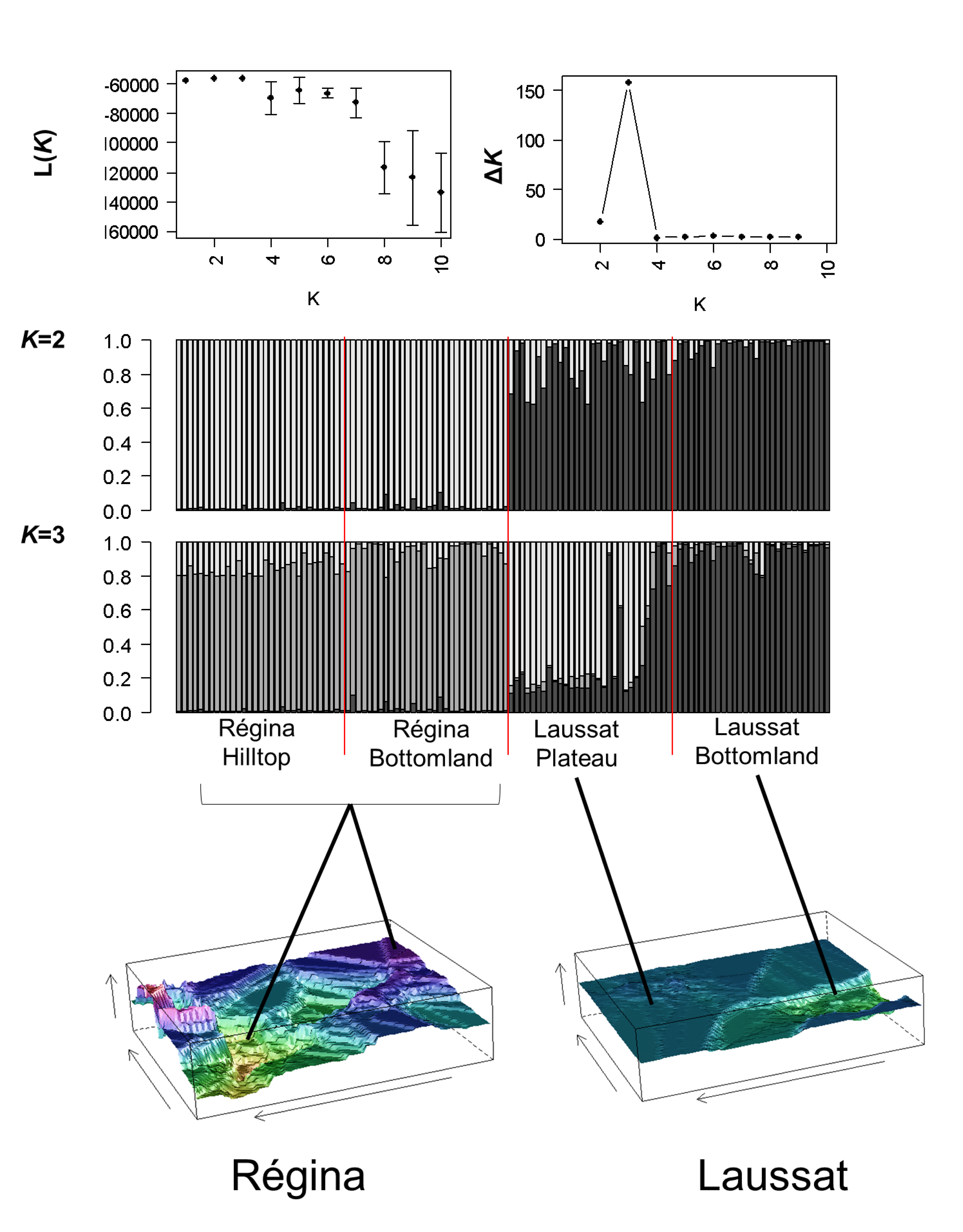

Supplement: S2 Fig — Upper pane: L(K) and ΔK values. Middle pane: individual α values for K = 2 and K = 3. Lower pane: geographical distribution of individuals belonging to the main clusters (see text). (TIF) [file pone.0121394.s002.tif]

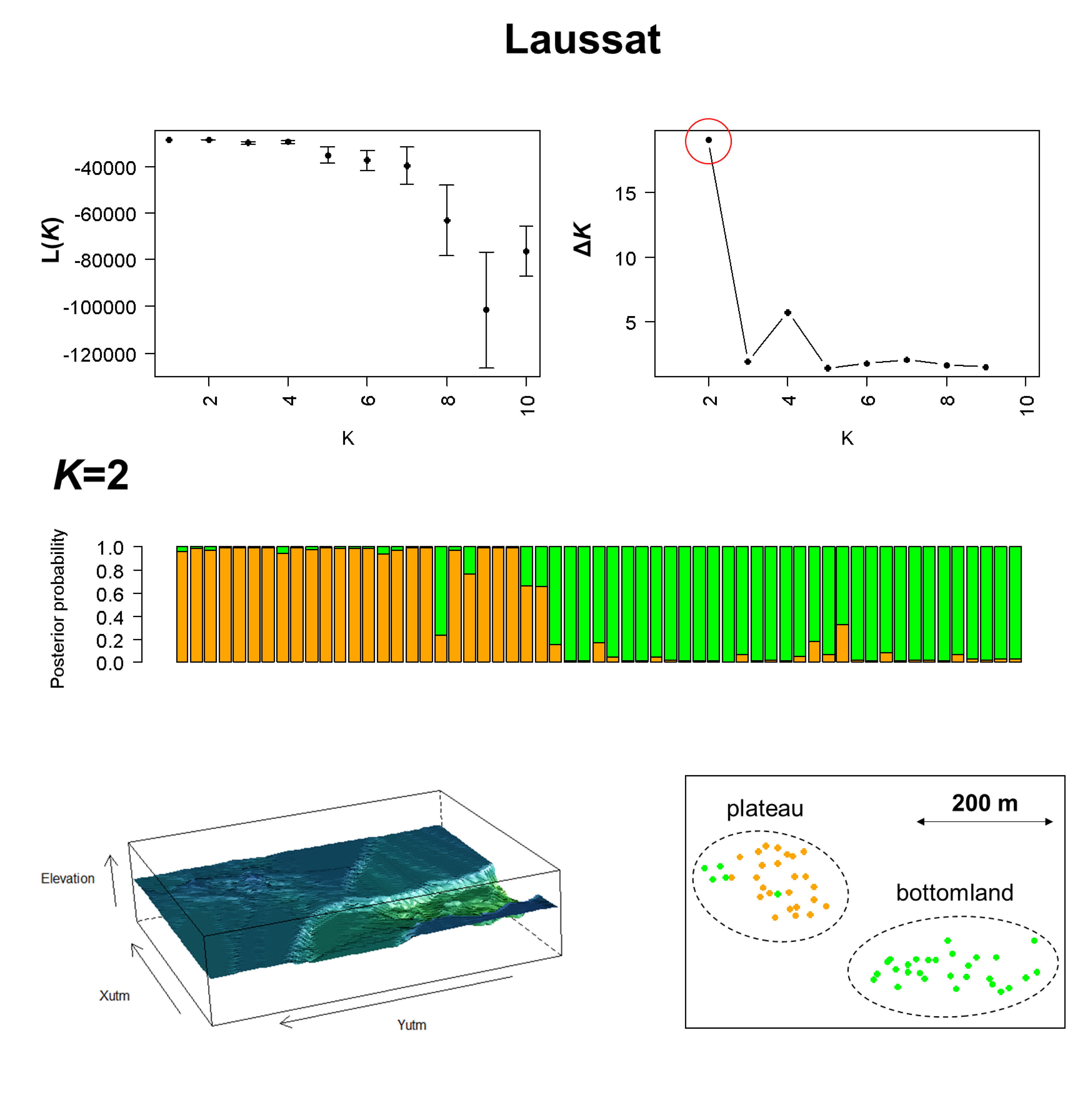

Supplement: S3 Fig — Upper pane: L(K) and ΔK values. Middle pane: individual α values for K = 2. Lower pane: geographical distribution of individuals belonging to the main clusters (see text). (TIF) [file pone.0121394.s003.tif]

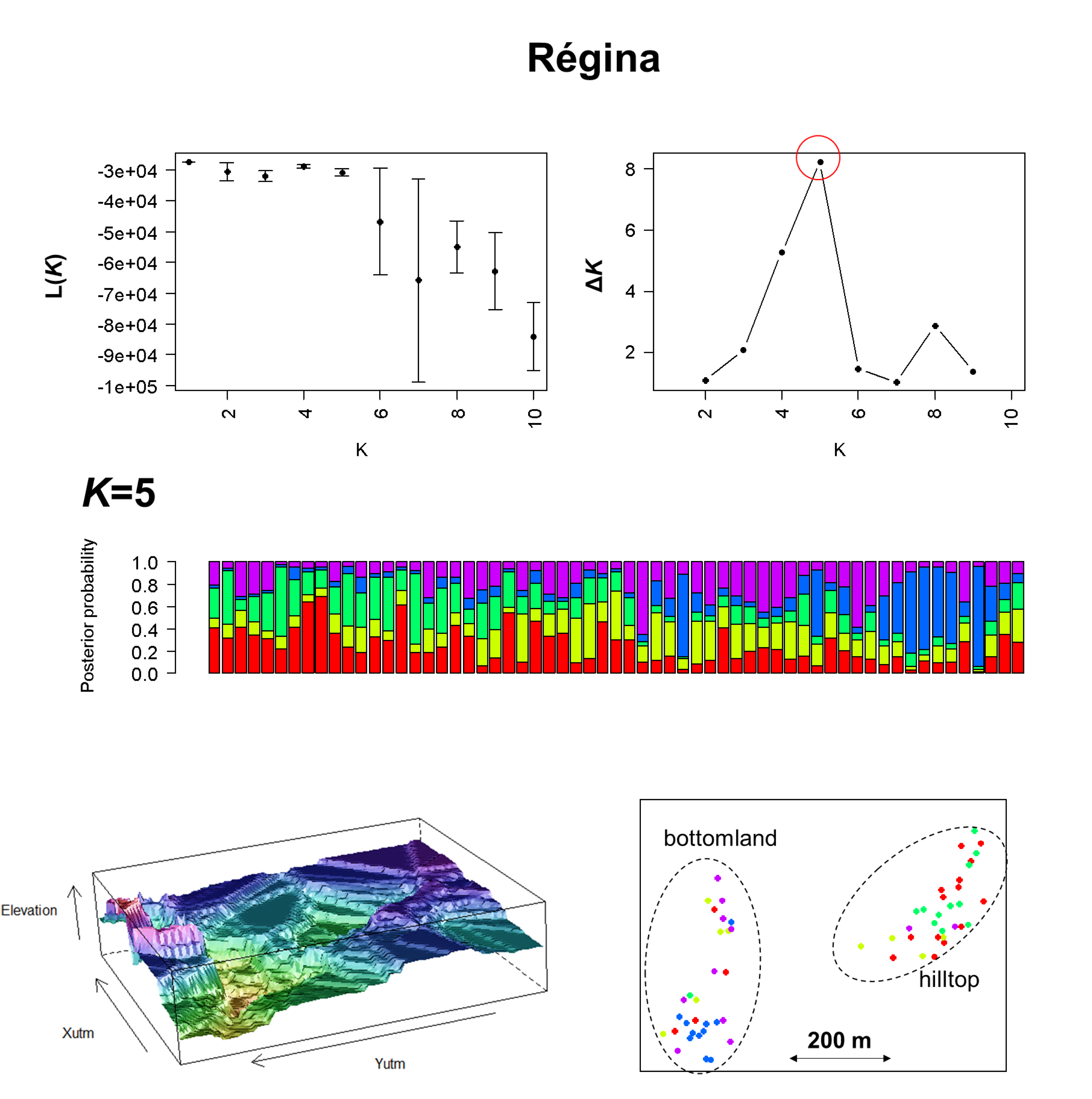

Supplement: S4 Fig — Upper pane: L(K) and ΔK values. Middle pane: individual α values for K = 5. Lower pane: geographical distribution of individuals belonging to the main clusters (see text). (TIF) [file pone.0121394.s004.tif]

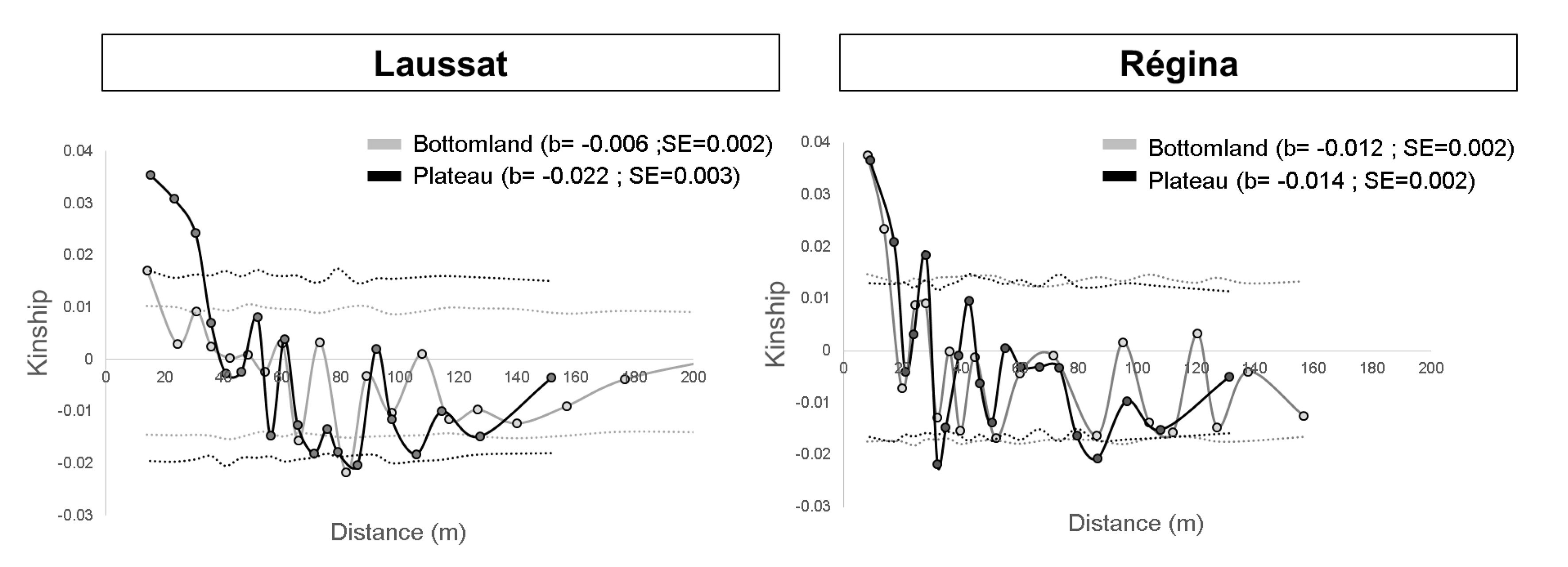

Supplement: S5 Fig — (TIF) [file pone.0121394.s005.tif]

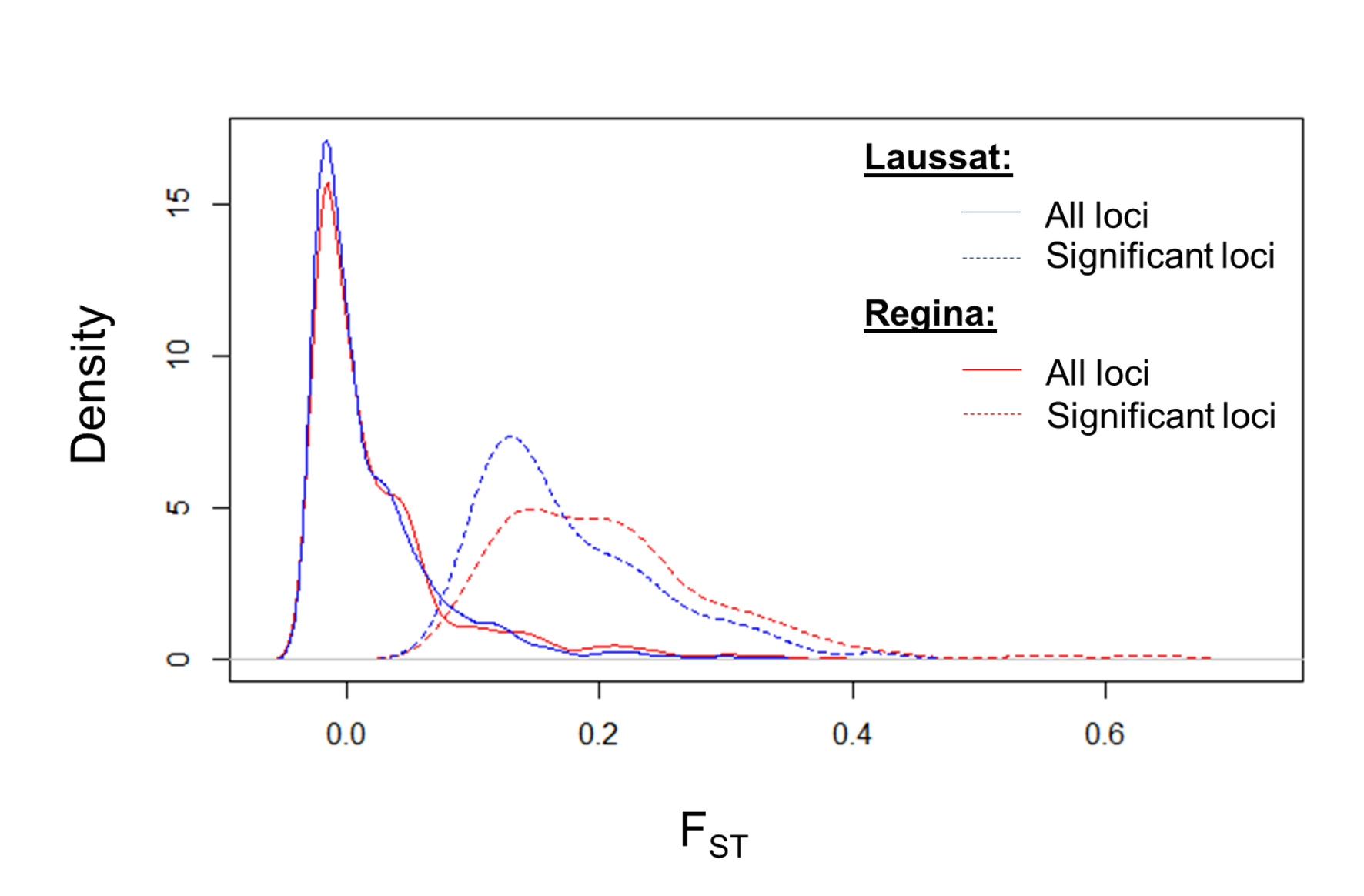

Supplement: S6 Fig — (TIF) [file pone.0121394.s006.tif]
