# Supplementary material for: Neutral and Adaptive Drivers of Microgeographic Genetic Divergence within Continuous Populations: The Case of the Neotropical Tree Eperua falcata (Aubl.)
Source: PLoS One. 2015 Mar 25;10(3):e0121394. doi: 10.1371/journal.pone.0121394 (PMC4373894; doi:10.1371/journal.pone.0121394)
Supplement: S1 Method — (DOCX) [file pone.0121394.s007.docx]

**Supplementary method S1.** AFLP scoring

**1-Reading of Peak profiles**

We used PeakScanner to read peak profiles within the range 50-500 bp.

**2-Thresholds definition**

We analyzed peaks profiles in both negative controls and sample profiles in the whole analysis window (50-500 bp) for defining detection thresholds. For each combination, we analyzed all peaks contained in negative controls and defined “lim.max.0” as the 95% quantile of peaks height within negative control. Thus, we suggest that the 5% of higher peaks in negative control may be “true peaks” due to contaminations, while 95% of peaks contained in negative controls may be considered as “true” background noise.

Then, we analyzed the distribution of all peaks higher than “lim.max.0” in sample profiles and defined a “lim.min.1” threshold corresponding to 25% of the distribution of peaks height outside background noise. These thresholds will be used to score “peak presence” in the further steps.


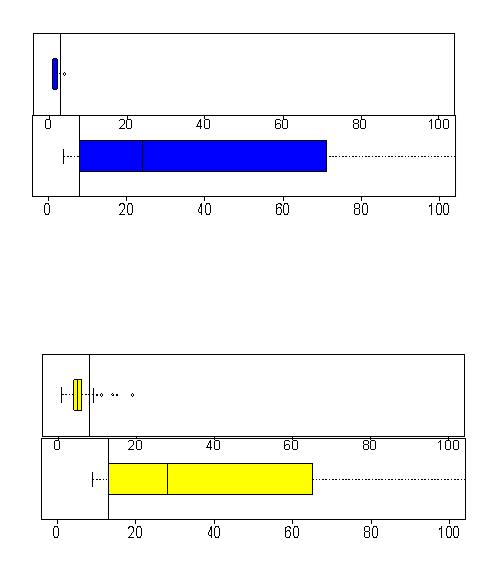


**Figure**: Peaks distribution and thresholds in both negative controls (top) and samples profiles (bottom)

**3- Binset definition**

Binset was defined using RawGeno (Arrigo 2009) with parameters: maximum=2bp, minimum bin width=1bp, range=50-500bp. We used “lim.min.1” as threshold for bin design. We didn’t use the “replicate” option because we wanted to analyze the replicates of each sample independently before doing consensus. Binset was manually corrected and exported. Last, we searched for peaks within bins and used the intensity of each sample within each bin for data cleaning and scoring steps.

**4. Data Pre-cleaning**

We pre-cleaned data by removing, for each combination, samples for which the two replicates were not available due to problems during genotyping (off-scale size standard or profile of bad quality).

**5. Scoring and consensus**

We assigned “0”, “N”, or “1” to each peak according with the criterions:

- Peak ≤ lim.max.0 : “0”
- lim.max.0 < Peak < lim.min.1 : “N”
- Peak ≥ lim.min.1 : “1”

and defined consensus as follow:

- 0/0 or 0/N : “0” (we considered the phenotype “0” if the replicates displayed two peaks within background noise or one peak within background noise and a “small peak” of intensity lower than lim.min.1).
- 1/1: “1”
- 1/N : “N” or “1” if a small peak outside blank (“N”) was supported by a peak of high intensity in the replicate (peak intensity within the 0.5 upper quantile of peaks intensity distribution within samples, see above).
- 0/1: “*” (“*” indicated a mismatch)
- N/N : “$” : (“$” indicated an ambiguity, i.e. the two replicates displayed two peaks outside background noise but intensity lower than lim.min.1)

**6. Data post-cleaning**

Data were post-cleaned by eliminating markers for which a peak of intensity higher than lim.min.1 was found in a least one negative control (contaminant).

We also remarked that peak intensity decreased within the 50-500bp window and that it was variable among profiles. It resulted in numerous 0 and missing data (including “N”, “*” and “$”) in profile tails that would result in assigning false-“0” to an absence of peak. To avoid it,

- We masked all “0” at the end of each profile (“N”) until a true peak (noted “1”) was found
- We removed all markers displaying any “1” or any “0”.
- We removed the last markers for which the proportion of “0” plus missing values (including “N”, “*” and “$”) were higher than the mean proportion of “0” plus missing values in bins of the whole dataset.


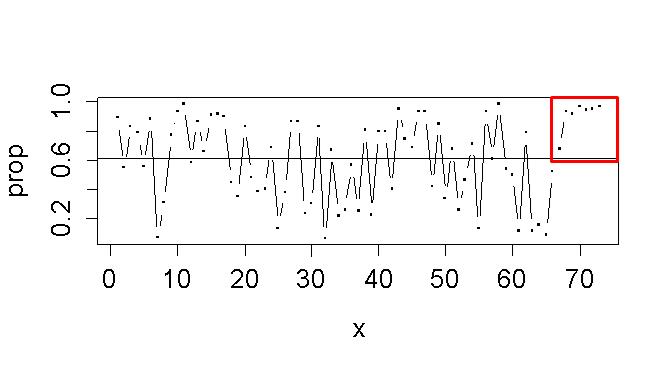


Last, we removed all markers that did not display at last 15 “true values” (true “0” or true “1”) for each site and local habitat.

**7- Data fusion**

Last we merged datasets from the different combinations. Samples that were absent from a combination (because one of the two replicates failed) were noted NA (by opposition with missing values due to post-treatment “N”, “*”, and “$”).
