# Supplementary material for: Neutral and Adaptive Drivers of Microgeographic Genetic Divergence within Continuous Populations: The Case of the Neotropical Tree Eperua falcata (Aubl.)
Source: PLoS One. 2015 Mar 25;10(3):e0121394. doi: 10.1371/journal.pone.0121394 (PMC4373894; doi:10.1371/journal.pone.0121394)
Supplement: S2 Method — (DOCX) [file pone.0121394.s008.docx]

**Supplementary method S2.** Model description and BUGS Code

**1. Model description**

In a first step, geographic, environmental and genetic distances matrices were drawn for all individual pairs. Genome-wide genetic distance (GENET) between individuals was computed from AFLPs data using the Jaccard’s distance (i.e. the number of loci for which the individuals have different AFLP phenotype, band presence or absence, divided by the total number of loci shared by the two individuals, i.e. non-missing for both). Geographic distances are described at regional scale by the membership of the individuals to different study sites (SITE), and at local scale by the continuous distance between two individuals based on their two-dimensional coordinates in a x,y-plane (GEO) and on their one-dimensional coordinates along an elevation gradient (ELEV) respectively. According to the environmental variations between the study sites and their local habitats, a discrete level of stress was attributed to each sites and local habitat for each of the environmental factor tested as following: SOIL TYPE: 1=ferralitic, 2=hygromorphic ; WATER-LOGGING: 1=no water-logging, 2=seasonal water-logging, 3=permanent water-logging ; SEASONAL SOIL DROUGHT: 1=low, 2=intermediate, 3=strong, 4=very strong. Because light and soil temperature were poorly variable among sites and local habitats, these two factors were excluded from the model. Environmental distances correspond to the absolute difference between the levels of stress experienced by each pair of individuals for the environmental factors tested (SOILTYPE, WATERLOG, DROUGHT).

In a second step, the genome-wide genetic distance for all individual pairs was partitioned according to the linear model:

**GENET_i1,i2_~Normal(meanGENET_i1,i2_,τ_R_)**

**meanGENET_i1,i2_=µ + (θ1 × SITE _i1,i2_) + (1- SITE_i1,i2_) × [(θ2 × GEO _i1,i2_) + (θ3 × ELEV_i1,i2_) ]+ (θ4 × DROUGHT_i1,i2_)+ (θ5 × WATERLOG_i1,i2_) + (θ6 × SOILTYPE_i1,i2_)**

µ is the global mean, τ_R_ is the residual precision (1/σ²_R_).

SITE_i1,i2_ traduces whether the individuals inhabit different study site (SITE_i1,i2_=0 if the individuals inhabit the same site, 1 if they inhabit different sites).

GEO _i1,i2_ and ELEV_i1,i2_ are both continuous variables traducing the local geographic distance within sites in the x,y-plane and along the elevation gradient respectively. Notice that (1- SITE_i1,i2_) is 1 if the individuals inhabit the same site, meaning that the following θ2 and θ3 parameters are estimated using pairs of individuals inhabiting the same site only.

DROUGHT_i1,i2_, WATERLOG_i1,i2_, and SOILTYPE_i1,i2_, are discrete variables describing the environmental distance between individuals for discrete variables (equal to 0 if they inhabit the same conditions).

The inferred parameters (θ1 to θ6) traduce the respective (and simultaneous) effects of geographic and environmental distances on the genetic distance between the analyzed individuals. Non-informative priors were used to infer the different parameters. These parameters has to be interpreted in terms of deviance from the global mean (μ). θ1 catches the effect of inhabiting different study site on the genetic distance between individuals. Θ2 and Θ3 catch intra-site neutral divergence as they traduce the slope of the relationship between geographic and genetic distance within site. θ4, θ5 and θ6 traduce adaptive divergence caused by the environmental conditions: they catch the effects of the environmental distance for discrete environmental variables (seasonal drought strength, water-logging frequency and soil type) on genome-wide genetic distance between individuals (all sites confounded).

**2. BUGS Code**

model{

for (i in 1:npairs)

{

DISTGENET[i]~dnorm(mean[i],tauR)

mean[i]<-mu+

(theta1*DIFSITE[i])+

(1-DIFSITE[i])*((theta2*DISTGEO[i])+(theta3*DIFELEV[i]))+

(theta4*DROUGHT[i])+

(theta5*WATERLOG[i])+

(theta6*SOILTYPE[i])

}

# priors

tauR~dgamma(0.01,0.01)

mu~dnorm(0,0.0001)

theta1~dnorm(0,0.0001)

theta2~dnorm(0,0.0001)

theta3~dnorm(0,0.0001)

theta4~dnorm(0,0.0001)

theta5~dnorm(0,0.0001)

theta6~dnorm(0,0.0001)

}
