# Supplementary material for: Neutral and Adaptive Drivers of Microgeographic Genetic Divergence within Continuous Populations: The Case of the Neotropical Tree Eperua falcata (Aubl.)
Source: PLoS One. 2015 Mar 25;10(3):e0121394. doi: 10.1371/journal.pone.0121394 (PMC4373894; doi:10.1371/journal.pone.0121394)
Supplement: S1 Table — (DOCX) [file pone.0121394.s009.docx]

**Supplementary Table S1.** Environmental conditions in each of the study sites and local habitat: soil type, water-logging frequency, soil humidity (‘Humidity’, %) and soil temperature (‘Temperature’, °C) at the end of the dry season, canopy opening (‘CnpyOpen’, %), leaf area index (‘LAI’, Mols.m-2.d-1, and total light transmitted to the ground (‘%TransTot’, %). Environmental conditions were characterized in 12 areas (~400 m²) located in each local habitat and study site (corresponding to 'experimental gardens' where a reciprocal transplant experiment is currently underway).

|  | Date | **Laussat** | **Laussat** | **Régina** | **Régina** |
| --- | --- | --- | --- | --- | --- |
|  |  | **Terra-firme** | **Bottomland** | **Terra-firme** | **Bottomland** |
| **Soil Type** | X | ferralitic | hygromorphic | ferralitic | hygromorphic |
| **Water-logging** | X | no | permanent | no | seasonal |
| **Humidity (%)** | 19-22/09/2011 | 7.6 | 100 | 17.4 | 41.6 |
| **Humidity (%)** | 19-22/09/2011 | 9.1 | 100 | 7.2 | 42.2 |
| **Humidity (%)** | 19-22/09/2011 | 7.2 | 100 | 21.2 | 37.8 |
| **Humidity (%)** | 19-22/09/2011 | 8.3 | 100 | 18.4 | 32.4 |
| **Humidity (%)** | 19-22/09/2011 | 8.6 | 100 | 10.7 | 29 |
| **Humidity (%)** | 19-22/09/2011 | 7.6 | 100 | 8.1 | 20.7 |
| **Humidity (%)** | 19-22/09/2011 | 7.4 | 94.8 | 21.1 | 26.9 |
| **Humidity (%)** | 19-22/09/2011 | 6.9 | 100 | 6.8 | 28.6 |
| **Humidity (%)** | 19-22/09/2011 | 11.3 | 44.7 | 12.6 | 44.3 |
| **Humidity (%)** | 19-22/09/2011 | 12.6 | 35.6 | 25 | 45.9 |
| **Humidity (%)** | 19-22/09/2011 | 18.1 | 44.2 | 25.8 | 48.9 |
| **Humidity (%)** | 19-22/09/2011 | 11.3 | 34.7 | 27.4 | 51.4 |
| **Humidity (%)** | 19-22/09/2011 | 15.2 | 46.3 | 26.3 | 36.9 |
| **Humidity (%)** | 19-22/09/2011 | 11.1 | 44.4 | 17.9 | 43.6 |
| **Humidity (%)** | 19-22/09/2011 | 11.4 | 39.3 | 24.9 | 33.9 |
| **Humidity (%)** | 19-22/09/2011 | 8.8 | 41 | 23.3 | 34 |
| **Humidity (%)** | 19-22/09/2011 | 15.2 | 46.9 | 6 | 34 |
| **Humidity (%)** | 19-22/09/2011 | 14.3 | 50.1 | 4.3 | 48.1 |
| **Humidity (%)** | 19-22/09/2011 | 21.2 | 44.2 | 6.5 | 32.9 |
| **Humidity (%)** | 19-22/09/2011 | 22.5 | 35.6 | 9.9 | 38.1 |
| **Humidity (%)** | 19-22/09/2011 | 13.3 | 37.1 | 5.5 | 43.1 |
| **Humidity (%)** | 19-22/09/2011 | 17 | 56.4 | 7.6 | 39.1 |
| **Humidity (%)** | 19-22/09/2011 | 18.3 | 48.2 | 7.7 | 29.1 |
| **Humidity (%)** | 19-22/09/2011 | 14.7 | 100 | 5.2 | 47.1 |
| **Humidity (%)** | 19-22/09/2011 | 7.4 | 100 | 18.1 | 36.5 |
| **Humidity (%)** | 19-22/09/2011 | 7.6 | 100 | 11.2 | 33.2 |
| **Humidity (%)** | 19-22/09/2011 | 8.6 | 100 | 13.6 | 40.8 |
| **Humidity (%)** | 19-22/09/2011 | 9.1 | 92.5 | 9.7 | 35.4 |
| **Humidity (%)** | 19-22/09/2011 | 8.5 | 67.3 | 9.1 | 35.2 |
| **Humidity (%)** | 19-22/09/2011 | 10.5 | 100 | 20 | 31.9 |
| **Humidity (%)** | 19-22/09/2011 | 7.7 | 97 | 7 | 34.6 |
| **Humidity (%)** | 19-22/09/2011 | 6.5 | 38.8 | 11.6 | 30.2 |
| **Humidity (%)** | 19-22/09/2011 | 11.8 | 13 | 28 | 39.5 |
| **Humidity (%)** | 19-22/09/2011 | 11.6 | 34.8 | 26.8 | 57.9 |
| **Humidity (%)** | 19-22/09/2011 | 17 | 35.3 | 28 | 39.2 |
| **Humidity (%)** | 19-22/09/2011 | 13.1 | 34.3 | 29.3 | 39 |
| **Humidity (%)** | 19-22/09/2011 | 8.7 | 57.6 | 25.6 | 37.1 |
| **Humidity (%)** | 19-22/09/2011 | 11.40 | 39.3 | 14.1 | 49.1 |
| **Humidity (%)** | 19-22/09/2011 | 9.8 | 45.2 | 24 | 38.1 |
| **Humidity (%)** | 19-22/09/2011 | 11.6 | 40.3 | 20.1 | 35.6 |
| **Humidity (%)** | 19-22/09/2011 | 16.4 | 37.2 | 7 | 35.4 |
| **Humidity (%)** | 19-22/09/2011 | 20.3 | 39.2 | 8.2 | 46.8 |
| **Humidity (%)** | 19-22/09/2011 | 27.6 | 39.9 | 4.1 | 33.4 |
| **Humidity (%)** | 19-22/09/2011 | 15.2 | 41.5 | 7 | 38.4 |
| **Humidity (%)** | 19-22/09/2011 | 12.8 | 37.5 | 5.2 | 42.1 |
| **Humidity (%)** | 19-22/09/2011 | 19.6 | 61.8 | 6.1 | 44.4 |
| **Humidity (%)** | 19-22/09/2011 | 19.9 | NA | 6 | 33.5 |
| **Humidity (%)** | 19-22/09/2011 | 13.2 | NA | 8 | 29.1 |
| **mean Humidity** | 19-22/09/2011 | 12.60 | 60.78 | 14.47 | 38.04 |
| **sd Humidity** | 19-22/09/2011 | 4.84 | 28.05 | 8.29 | 7.15 |
| **Humidity (%)** | 3-9/12/2012 | 9.34 | 68.97 | 0 | 31.28 |
| **Humidity (%)** | 3-9/12/2012 | 12.64 | 54.86 | 5.28 | 37.13 |
| **Humidity (%)** | 3-9/12/2012 | 10.51 | 86.73 | 3.7 | 38.17 |
| **Humidity (%)** | 3-9/12/2012 | 11.74 | 41.35 | 0 | 21.56 |
| **Humidity (%)** | 3-9/12/2012 | 6.91 | 49.02 | 0 | 26.99 |
| **Humidity (%)** | 3-9/12/2012 | 6.36 | 95.86 | 0 | 24.61 |
| **Humidity (%)** | 3-9/12/2012 | 6.73 | 69.43 | 0 | 22.88 |
| **Humidity (%)** | 3-9/12/2012 | 6.11 | 50.83 | 0 | NA |
| **Humidity (%)** | 3-9/12/2012 | 9.99 | 36.77 | 4.89 | 21.71 |
| **Humidity (%)** | 3-9/12/2012 | 17 | 32.32 | 0.74 | 33.11 |
| **Humidity (%)** | 3-9/12/2012 | 15.79 | 44.47 | 4.63 | 35.27 |
| **Humidity (%)** | 3-9/12/2012 | 8.27 | 34.91 | 0 | 16.3 |
| **Humidity (%)** | 3-9/12/2012 | 13.93 | 39.17 | 0 | 23.76 |
| **Humidity (%)** | 3-9/12/2012 | 15.93 | 53.24 | 2.88 | 40.76 |
| **Humidity (%)** | 3-9/12/2012 | 9.68 | 39.6 | 6.58 | 41.53 |
| **Humidity (%)** | 3-9/12/2012 | 15.55 | 38.78 | 3.61 | 32.29 |
| **Humidity (%)** | 3-9/12/2012 | 23.18 | 29.94 | 1.54 | 35.47 |
| **Humidity (%)** | 3-9/12/2012 | 14.66 | 42.18 | 0 | 53.61 |
| **Humidity (%)** | 3-9/12/2012 | 14.6 | 43.57 | 1.32 | 33.39 |
| **Humidity (%)** | 3-9/12/2012 | 13.62 | 38.33 | 0.88 | 36.36 |
| **Humidity (%)** | 3-9/12/2012 | 17.42 | 48.43 | 0.33 | 45.44 |
| **Humidity (%)** | 3-9/12/2012 | 11.82 | 39.47 | 0 | 38.78 |
| **Humidity (%)** | 3-9/12/2012 | 7.71 | 74.14 | 0 | 30.72 |
| **Humidity (%)** | 3-9/12/2012 | 8.01 | NA | 0 | 29.59 |
| **mean Humidity** | 3-9/12/2012 | 11.98 | 50.10 | 1.52 | 32.64 |
| **sd Humidity** | 3-9/12/2012 | 4.32 | 17.50 | 2.11 | 8.69 |
| **Temperature (°C)** | 3-9/12/2012 | 27.5 | 23.9 | 29.36 | 31 |
| **Temperature (°C)** | 3-9/12/2012 | 27.5 | 24.3 | 29.5 | 31 |
| **Temperature (°C)** | 3-9/12/2012 | 27.5 | 24.2 | 29.5 | 30.9 |
| **Temperature (°C)** | 3-9/12/2012 | 27.5 | 24.1 | 29.4 | 31 |
| **Temperature (°C)** | 3-9/12/2012 | 27.5 | 24.1 | 29.4 | 31 |
| **Temperature (°C)** | 3-9/12/2012 | 27.5 | 24.3 | 29.5 | 30.9 |
| **Temperature (°C)** | 3-9/12/2012 | 27.6 | 24.1 | 29.6 | 31 |
| **Temperature (°C)** | 3-9/12/2012 | 27.5 | 24 | 29.5 | NA |
| **Temperature (°C)** | 3-9/12/2012 | 30.4 | 24.5 | 29 | 29.6 |
| **Temperature (°C)** | 3-9/12/2012 | 30.6 | 24.4 | 29.2 | 29.6 |
| **Temperature (°C)** | 3-9/12/2012 | 30.5 | 24.5 | 29 | 29.5 |
| **Temperature (°C)** | 3-9/12/2012 | 30.4 | 24.5 | 29.3 | 29.6 |
| **Temperature (°C)** | 3-9/12/2012 | 30.5 | 24.5 | 29.2 | 29.6 |
| **Temperature (°C)** | 3-9/12/2012 | 30.5 | 24.5 | 29.1 | 29.6 |
| **Temperature (°C)** | 3-9/12/2012 | 30.3 | 24.6 | 29.1 | 29.7 |
| **Temperature (°C)** | 3-9/12/2012 | 30.5 | 24.6 | 29.4 | 29.7 |
| **Temperature (°C)** | 3-9/12/2012 | 27.9 | 22.3 | 28.6 | 29.2 |
| **Temperature (°C)** | 3-9/12/2012 | 28.1 | 22 | 28.9 | 29.3 |
| **Temperature (°C)** | 3-9/12/2012 | 27.9 | 22 | 28.6 | 29.1 |
| **Temperature (°C)** | 3-9/12/2012 | 28 | 21.9 | 28.8 | 29.3 |
| **Temperature (°C)** | 3-9/12/2012 | 28 | 22 | 28.8 | 29.3 |
| **Temperature (°C)** | 3-9/12/2012 | 28 | 22 | 28.7 | 29.1 |
| **Temperature (°C)** | 3-9/12/2012 | 28 | 22.2 | 28.7 | 29.2 |
| **Temperature (°C)** | 3-9/12/2012 | 28 | NA | 28.8 | 29.3 |
| **mean T°** | 3-9/12/2012 | 28.65 | 23.63 | 29.12 | 29.89 |
| **sd T°** | 3-9/12/2012 | 9.17 | 18.08 | 14.00 | 6.94 |
| **%CnpyOpen** | 10/12/2012 | 7.19 | 5.44 | 12.59 | 6.38 |
| **%CnpyOpen** | 10/12/2012 | 6.92 | 5.66 | 7.04 | 5.66 |
| **%CnpyOpen** | 10/12/2012 | 6.59 | 6.22 | 8.08 | 6.71 |
| **%CnpyOpen** | 10/12/2012 | 6.52 | 5.82 | 8.19 | 7.34 |
| **%CnpyOpen** | 10/12/2012 | 5.59 | 6.57 | 7.84 | 5.95 |
| **%CnpyOpen** | 10/12/2012 | 7.04 | 7.44 | 8.56 | 5.8 |
| **%CnpyOpen** | 10/12/2012 | 8.5 | 7.21 | 7.37 | 7.48 |
| **%CnpyOpen** | 10/12/2012 | 8.95 | 8.41 | 8.47 | 6.2 |
| **%CnpyOpen** | 10/12/2012 | 7.61 | 6.6 | 8.45 | 9.46 |
| **%CnpyOpen** | 10/12/2012 | 8.14 | 5.83 | 8.13 | 7.5 |
| **%CnpyOpen** | 10/12/2012 | 6.55 | NA | 6.9 | 5.78 |
| **%CnpyOpen** | 10/12/2012 | 6.55 | NA | 7.15 | 5.36 |
| **%CnpyOpen** | 10/12/2012 | 7.14 | NA | 7.1 | 7.58 |
| **%CnpyOpen** | 10/12/2012 | 7.28 | NA | 6.84 | 5.75 |
| **%CnpyOpen** | 10/12/2012 | 6.49 | NA | 6.28 | 5.35 |
| **%CnpyOpen** | 10/12/2012 | NA | NA | NA | 7.32 |
| **%CnpyOpen** | 10/12/2012 | NA | NA | NA | 7.68 |
| **%CnpyOpen** | 10/12/2012 | NA | NA | NA | 8.25 |
| **%CnpyOpen** | 10/12/2012 | NA | NA | NA | 6.83 |
| **%CnpyOpen** | 10/12/2012 | NA | NA | NA | 6.69 |
| **mean %CnpyOpen** | 10/12/2012 | 7.14 | 6.52 | 7.937 | 6.75 |
| **sd CnpyOpen** | 10/12/2012 | 0.87 | 0.93 | 1.47 | 1.07 |
| **LAI (Mols.m-2.d-1)** | 10/12/2012 | 2.945 | 3.3 | 2.225 | 3.24 |
| **LAI (Mols.m-2.d-1)** | 10/12/2012 | 2.925 | 3.26 | 2.89 | 3.335 |
| **LAI (Mols.m-2.d-1)** | 10/12/2012 | 3.12 | 3.035 | 2.75 | 3.425 |
| **LAI (Mols.m-2.d-1)** | 10/12/2012 | 3.125 | 3.19 | 2.81 | 2.87 |
| **LAI (Mols.m-2.d-1)** | 10/12/2012 | 3.28 | 2.95 | 2.815 | 3.21 |
| **LAI (Mols.m-2.d-1)** | 10/12/2012 | 2.88 | 2.765 | 2.65 | 3.175 |
| **LAI (Mols.m-2.d-1)** | 10/12/2012 | 2.6 | 2.84 | 2.785 | 2.85 |
| **LAI (Mols.m-2.d-1)** | 10/12/2012 | 2.58 | 2.6 | 2.65 | 3.255 |
| **LAI (Mols.m-2.d-1)** | 10/12/2012 | 2.78 | 2.96 | 2.63 | 2.545 |
| **LAI (Mols.m-2.d-1)** | 10/12/2012 | 2.68 | 3.06 | 2.71 | 2.865 |
| **LAI (Mols.m-2.d-1)** | 10/12/2012 | 3 | NA | 2.93 | 3.315 |
| **LAI (Mols.m-2.d-1)** | 10/12/2012 | 3.07 | NA | 2.895 | 3.43 |
| **LAI (Mols.m-2.d-1)** | 10/12/2012 | 2.89 | NA | 2.945 | 2.995 |
| **LAI (Mols.m-2.d-1)** | 10/12/2012 | 2.85 | NA | 2.995 | 3.305 |
| **LAI (Mols.m-2.d-1)** | 10/12/2012 | 3.005 | NA | 3.125 | 3.405 |
| **LAI (Mols.m-2.d-1)** | 10/12/2012 | NA | NA | NA | 2.99 |
| **LAI (Mols.m-2.d-1)** | 10/12/2012 | NA | NA | NA | 3.035 |
| **LAI (Mols.m-2.d-1)** | 10/12/2012 | NA | NA | NA | 2.98 |
| **LAI (Mols.m-2.d-1)** | 10/12/2012 | NA | NA | NA | 3.51 |
| **LAI (Mols.m-2.d-1)** | 10/12/2012 | NA | NA | NA | 3.085 |
| **mean LAI** | 10/12/2012 | 2.92 | 3.00 | 2.79 | 3.14 |
| **sd LAI** | 10/12/2012 | 0.20 | 0.22 | 0.21 | 0.25 |
| **%TransToT** | 10/12/2012 | 12.76 | 8.93 | 15.86 | 9.43 |
| **%TransToT** | 10/12/2012 | 12.16 | 10.14 | 9.04 | 10.49 |
| **%TransToT** | 10/12/2012 | 11.49 | 8.8 | 10.62 | 12.16 |
| **%TransToT** | 10/12/2012 | 10.95 | 9.26 | 11.49 | 11.22 |
| **%TransToT** | 10/12/2012 | 11.45 | 10.28 | 11.99 | 9.94 |
| **%TransToT** | 10/12/2012 | 11.29 | 9.83 | 10.75 | 9.97 |
| **%TransToT** | 10/12/2012 | 13 | 10.83 | 11.05 | 10.63 |
| **%TransToT** | 10/12/2012 | 13.47 | 10.84 | 10.55 | 10.02 |
| **%TransToT** | 10/12/2012 | 12.12 | 11.06 | 10.66 | 15.25 |
| **%TransToT** | 10/12/2012 | 11.58 | 8.67 | 10.24 | 11.38 |
| **%TransToT** | 10/12/2012 | 10.39 | NA | 10.17 | 9.93 |
| **%TransToT** | 10/12/2012 | 12.55 | NA | 11.1 | 8.99 |
| **%TransToT** | 10/12/2012 | 12.63 | NA | 12.78 | 14 |
| **%TransToT** | 10/12/2012 | 12.65 | NA | 11.16 | 10.6 |
| **%TransToT** | 10/12/2012 | 10.63 | NA | 10.35 | 9.36 |
| **%TransToT** | 10/12/2012 | NA | NA | NA | 15.3 |
| **%TransToT** | 10/12/2012 | NA | NA | NA | 15.56 |
| **%TransToT** | 10/12/2012 | NA | NA | NA | 15.28 |
| **%TransToT** | 10/12/2012 | NA | NA | NA | 15.68 |
| **%TransToT** | 10/12/2012 | NA | NA | NA | 13.69 |
| **mean TransToT** | 10/12/2012 | 11.94 | 9.864 | 11.19 | 11.94 |
| **sd TransToT** | 10/12/2012 | 0.91 | 0.90 | 1.55 | 2.42 |
