# Supplementary material for: Correction: Neutral and Adaptive Drivers of Microgeographic Genetic Divergence within Continuous Populations: The Case of the Neotropical Tree Eperua falcata (Aubl.)
Source: PLoS One. 2015 Apr 22;10(4):e0127134. doi: 10.1371/journal.pone.0127134 (PMC4406816; doi:10.1371/journal.pone.0127134)
Supplement: S2 File — (PDF) [file pone.0127134.s002.pdf]

RESEARCH ARTICLE

Neutral and Adaptive Drivers of Microgeographic Genetic Divergence within Continuous Populations: The Case of the Neotropical Tree *Eperua falcata* (Aubl.)

Louise Brousseau[1,2,3]*, Matthieu Foll[4,5], Caroline Scotti-Saintagne[1,3], Ivan Scotti[1,3]

1 INRA, UMR745 EcoFoG Ecologie des forêts de Guyane, Campus Agronomique BP316, 97379 Kourou Cedex, France, **2** INRA—Université de Lorraine, UMR1137 EEF Ecologie et Ecophysiologie Forestière, allée de l'Arboretum, 54280 Champenoux, France, **3** INRA, UR629 URFM Ecologie des Forêts Méditerranéennes, Domaine Saint Paul, Site Agroparc CS 40509, 84914 Avignon Cedex 9, France, **4** School of Life Sciences, Ecole Polytechnique Fédérale de Lausanne (EPFL), Station 15, CH-1015 Lausanne, Switzerland, **5** Swiss Institute of Bioinformatics, Lausanne, Switzerland

* louise.brousseau@ecofog.gf

OPEN ACCESS

Citation: Brousseau L, Foll M, Scotti-Saintagne C, Scotti I (2015) Neutral and Adaptive Drivers of Microgeographic Genetic Divergence within Continuous Populations: The Case of the Neotropical Tree *Eperua falcata* (Aubl.). PLoS ONE 10(3): e0121394. doi:10.1371/journal.pone.0121394

Academic Editor: Filippos A. Aravanopoulos, Aristotle University of Thessaloniki, GREECE

Received: October 21, 2014

Accepted: January 31, 2015

Published: March 25, 2015

Copyright: © 2015 Brousseau et al. This is an open access article distributed under the terms of the Creative Commons Attribution License, which permits unrestricted use, distribution, and reproduction in any medium, provided the original author and source are credited.

Data Availability Statement: Tree location, coordinates and AFLPs data (binary) are available on Dryad doi: 10.5061/dryad.b2q88.

Funding: This study was funded by the ANR-BIOADAPT FLAG program (ANR-12-ADAP-0007-01) and by an 'Investissement d'Avenir' grant (CEBA, ANR-10-LABX-0025), both managed by The French National Research Agency (ANR, France, www.agence-nationale-recherche.fr) and granted to Ivan Scotti and Caroline Scotti-Saintagne. Louise Brousseau's Ph.D. thesis and Post-doc were funded by a 'Young Scientist Contract' ('CJS') of the French

Abstract

Background

In wild plant populations, genetic divergence within continuous stands is common, sometimes at very short geographical scales. While restrictions to gene flow combined with local inbreeding and genetic drift may cause neutral differentiation among subpopulations, microgeographical variations in environmental conditions can drive adaptive divergence through natural selection at some targeted loci. Such phenomena have recurrently been observed in plant populations occurring across sharp environmental boundaries, but the interplay between selective processes and neutral genetic divergence has seldom been studied.

Methods

We assessed the extent of within-stand neutral and environmentally-driven divergence in the Neotropical tree *Eperua falcate* Aubl. (Fabaceae) through a genome-scan approach. Populations of this species grow in dense stands that cross the boundaries between starkly contrasting habitats. Within-stand phenotypic and candidate-gene divergence have already been proven, making this species a suitable model for the study of genome-wide microgeographic divergence. Thirty trees from each of two habitats (seasonally flooded swamps and well-drained plateaus) in two separate populations were genotyped using thousands of AFLPs markers. To avoid genotyping errors and increase marker reliability, each sample was genotyped twice and submitted to a rigorous procedure for data cleaning, which resulted in 1196 reliable and reproducible markers.

National Institute for Agronomical Research (INRA, France, www.inra.fr). The funders had no role in study design, data collection and analysis, decision to publish, or preparation of the manuscript.

Competing Interests: The authors have declared that no competing interests exist.

Results

Despite the short spatial distances, we detected within-populations genetic divergence, probably caused by neutral processes, such as restrictions in gene flow. Moreover, habitat-structured subpopulations belonging to otherwise continuous stands also diverge in relation to environmental variability and habitat patchiness: we detected convincing evidence of divergent selection at the genome-wide level and for a fraction of the analyzed loci (comprised between 0.25% and 1.6%). Simulations showed that the levels of differentiation for these outliers are compatible with scenarios of strong divergent selection.

Introduction

Microgeographic genetic divergence [1, 2] (i.e. the genetic divergence occurring within continuous populations over geographical scales in the same range as species' dispersal neighborhood, in spite of extensive gene flow) has been frequently demonstrated in plant populations, at least as early as the middle of the 20th century for both phenotypic traits [3–5] and molecular markers [1, 6–9]. Microgeographic divergence has been the subject of major review articles [10–12] arguing that adaptive processes are relatively widespread at these very local scales. However, Spatial Genetic Structure (SGS) is also common at local scales in wild plant populations [13, 14]. This commonly implies neutral divergence caused by restrictions in gene flow (pollen and seeds), genetic drift and mating processes (such as mating among neighbors and local inbreeding) [15, 16]. These processes are supposedly reinforced in plants because they are sessile, even more in trees because of their long life cycle and large progeny sizes [17–19]. Microgeographic neutral divergence is very common in tropical tree species [13, 20, 21], although the observed genetic structures are generally shallow. Pollen and seed flow are often restricted because air humidity and frequent precipitation prevent wind dispersion of pollen and seeds, and because the heavy seeds are often dispersed by gravity close to maternal tree crowns [16, 22], as it is the case in the bat-pollinated and autochorous canopy tree *Eperua falcata* Aublet (Fabaceae). Consequently, mating among neighbors is frequent in aggregative tree species, causing local inbreeding and contributing to the spatial genetic structuring [20].

While neutral genetic divergence is independent of habitat variation (except when such variation induces barriers to dispersal [23]), adaptive genetic divergence is driven by habitat transitions at least for some specific loci [8, 10, 23]. In this case, the genetic differentiation is expected to be stronger for adaptive loci than for neutral ones. This difference provides a theoretical framework for the discrimination of neutral and adaptive sources of microgeographic differentiation.

Amazonian lowland forests are characterized by complex habitat patchiness whereby environmental conditions vary at a small spatial scale (i.e. in the order of hundreds of meters). The succession of waterlogged bottomlands and well-drained '*terra firme*' plateaus is associated with strong variations in tree communities [24–27]. Microgeographic environmental variability is thus likely to participate to the maintenance of high diversity of tree species in the forest landscapes of Amazonia [28]. More precisely, it has been suggested that divergent selective pressures among local habitat types may have driven the specialization of trees species for local conditions, and that ecological divergences among congeneric species would result from adaptive radiations along topography gradients [29]. In *E. falcata*, a recent study has revealed footprints of divergent selection between local subpopulations occupying distinct habitats at

stress-response genes [8]. Genetic differentiation was accompanied by consistent phenotypic divergence for growth and leaf physiology at the seedling stage in *E. falcata* and in the congeneric *E. grandiflora* [30]. These preliminary results make *E. falcata* a good model for the analysis of adaptive processes over microgeographical scales.

In this study, we analyzed the neutral and adaptive sources of genetic structuring within continuous stands of *E. falcata* in the eastern Guiana shield (French Guiana). To achieve our goals, four populations (corresponding to the replication of the microgeographic 'hilltop *versus* bottomland' environmental contrast in two distinct stands) were scanned with approximately 1200 AFLP markers. Genome-wide spatial genetic structure was evaluated and the extent of genetic divergence was assessed at both regional and microgeographical scales. A landscape approach was combined with outlier detection tests to distinguish between neutral and adaptive sources of genetic divergence, and to determine whether microgeographic adaptation to local habitat patchiness was involved in genome-wide and/or locus-specific genetic divergence.

Methods

Ethics statements

E. falcata leaf samples were collected in two study sites of the Eastern Guiana shield: Laussat (5°28'37"N; -53°34'36"W) and Régina (4°18'44"N; -52°14'6"W). The study sites are managed by the French National Forests Office (ONF) which authorized tree labelling and leaf sampling. *E. falcata* is not a protected or endangered species and we certify that our experiment complies with the laws and ethical recommendations of France and French Guiana.

Species description, study sites and sampling

Eperua falcata (Aublet) is a canopy-subdominant tree species, hyper-abundant in the Guiana shield [31]. Its distribution is aggregative, and aggregates often reach high population densities. Pollination is ensured by bats while seed dispersal is autochorous [32]: heavy seeds are dispersed at short distance around crowns of mother trees through explosive dehiscence. Our study includes two *E. falcata* populations located near the coast of the Eastern Guiana shield: Laussat (5°28'37"N; -53°34'36"W) and Régina (4°18'44"N; -52°14'6"W). These populations experience contrasted rainfall regimes, with a mean annual precipitation of 2500 mm and 3500 mm respectively (in years 2010 and 2011), and with a harsher dry season in Laussat (data from météo-FRANCE stations of 'Iracoubo' and 'Régina'), Fig. 1. Both sites harbor different habitat types, from a bottomland to *terra firme*, and differ in landscape raggedness. In Laussat, a permanently water-logged bottomland gently rises toward a plateau of low elevation. In Régina, narrow seasonally flooded bottomlands and streambeds lie at the foot of hills and higher-elevation plateaus with steeper slopes. In both sites, bottomlands are characterized by hygromorphic soils with a large accumulation of organic matter up to a depth of 1 m caused by intense waterlogging, while *terra firme* are composed of well-drained ferralitic soils, rich in iron oxides with a sand-clay texture allowing free vertical drainage (S1 Fig.). Soil humidity and temperature were assessed at the end of the dry season (in 2011 and 2012) in each study site and local habitat using a soil moisture sensor TRIME-PICO32 (Table 1, Fig. 2 and S1 Table). Canopy opening was estimated by realizing fisheye hemispherical photographs with a Nikon digital camera and treated using Gap Light Analyzer V2.0 [33] to estimate canopy opening, Leaf Area Index (LAI) and the total light transmitted to ground (Fig. 2, S1 Fig., and S1 Table).

All trees of diameter at breast height (d.b.h.) > 20 cm were mapped in a continuous area of 6.7 ha in Régina, and in two areas of 2.5 ha and 1.8 ha in Laussat. Population density varied between 29.9 adult trees/ha and 48.11 trees/ha in Régina and Laussat respectively. In each site, two groups of 30 trees inhabiting distinct habitat types (named 'bottomland' and 'hilltop',

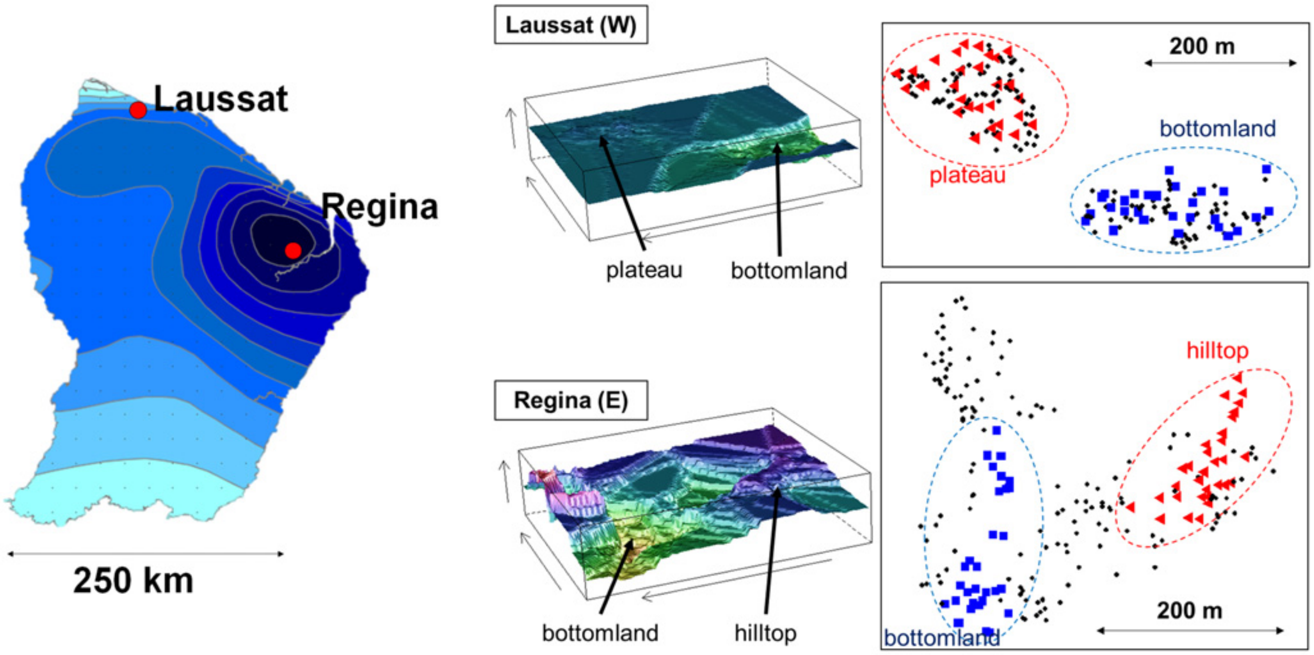

Fig 1. Geographic and topographic situation of the study sites. Colored dots: trees sampled for genotyping (triangles: hilltop; squares: bottomland).

doi:10.1371/journal.pone.0121394.g001

Fig. 1) were randomly selected and sampled for genetic analyses, totaling 120 trees with elevations ranging from 17 to 60 m a.s.l. in Laussat and from 47 to 92 m a.s.l in Régina. The sample size was set to 30 trees per population, in agreement with the intermediate sample size simulated by Foll and Gaggiotti [34] to test the powerful of their method to detect dominant loci under selection. Tree descriptions (site, local habitat, coordinates) are accessible on Dryad (http://dx.doi.org/10.5061/dryad.b2q88).

Molecular methods

Genome-scans are very powerful for apprehending the extent of genome-wide genetic differentiation in wild populations and for detecting locus-specific signatures of population divergence [35] which can be interpreted as suggestive of the action of natural selection [36]. In non-model species, AFLP markers [37] are widely used for genome-wide analyses of within-population genetic variation [38–43]. Despite the drawbacks of being dominant and anonymous, they have been proved to outperform other markers-such as micro-satellites- in the detection of genetic structure [44] and in the discrimination of taxa and populations [45, 46]. They present

Table 1. Summary of the environmental conditions in the study sites and local habitats: soil type, waterlogging frequency and seasonal soil drought severity.

Site and local habitat	Laussat Plateau	Laussat Bottomland	Régina Hilltop	Régina Bottomland
Soil Type	Ferralitic	Hygromorphic	Ferralitic	Hygromorphic
Waterlogging frequency	no	permanent	no	seasonal
Seasonal Soil Drought Severity	strong	low	very strong	intermediate

Complete data are provided in S1 Table (see also Fig. 2).

doi:10.1371/journal.pone.0121394.t001

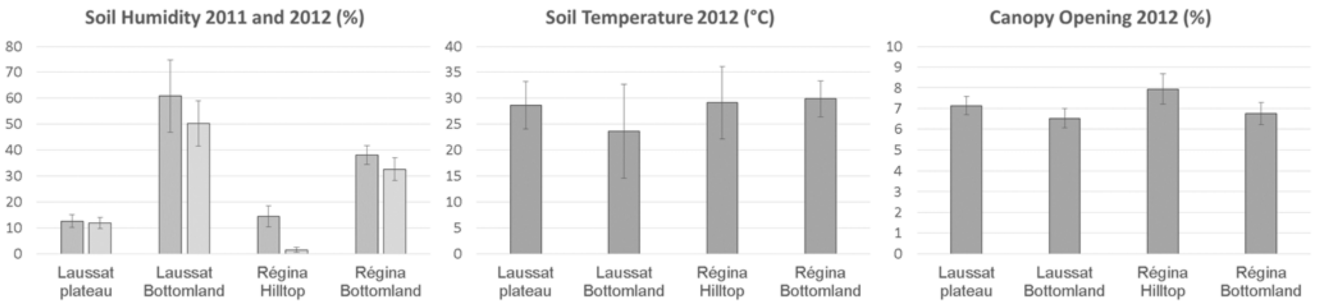

Fig 2. Environmental conditions in the study sites and local habitats: soil humidity (%), soil temperature (°C) and canopy opening (%). Complete data are provided in S1 Table.

doi:10.1371/journal.pone.0121394.g002

the obvious advantage of being easily obtained, relatively robust, and of requiring no prior sequence information [45–48]. These markers have, however, been largely criticized for their lack of reproducibility [49] and require rigorous strategies to check repeatability and to control for genotyping errors [50].

Fresh leaves were sampled and frozen at -80°C as soon as they arrived to the lab (in the evening following the sampling). Genomic DNA was extracted using a CTAB protocol [51, 52], and each sample was extracted twice independently. Amplified fragment length polymorphisms (AFLPs) profiling was performed according to the protocol of Vos, Hogers and Bleeker [37]. DNA was digested using *Pst*I and *Mse*I restriction enzymes [37, 53–55]. Restriction fragments were amplified through two selective PCRs with respectively one and three selective nucleotides. Fifteen primer combinations were analyzed: *Pst*+ACA/*Mse*+TAA, *Pst*+ATT/*Mse*+TAA, *Pst*+AAC/*Mse*+TAA, *Pst*+ATA/*Mse*+TAA, *Pst*+ACA/*Mse*+TAG, *Pst*+ATT/*Mse*+TAG, *Pst*+AAC/*Mse*+TAG, *Pst*+ATA/*Mse*+TAG, *Pst*+TAA/*Mse*+CAA, *Pst*+TAG/*Mse*+CAA, *Pst*+ACA/*Mse*+CAA, *Pst*+ATA/*Mse*+CAT, *Pst*+ACA/*Mse*+CAT, *Pst*+ATT/*Mse*+CAT, *Pst*+ATA/*Mse*+CAT. The complete protocol (including DNA extraction, AFLP protocol and genotyping) was realized twice independently for each sample to obtain a complete replicate of the dataset (totaling 2 x 120 trees = 240 samples).

AFLPs were scored through an automated cleaning procedure (encoded in R): (i) negative controls were used to define thresholds of peak detection, (ii) peak profiles were scanned using PeakScanner Software v1.0 (Applied Biosystems) and the bin set was created using RawGeno v2.0 [47] with the previously defined thresholds, (iii) a consensus AFLP profile was edited for each sampled tree (only well replicated genotypes were kept, genotypes that were not replicated were considered as missing), (iv) data were post-cleaned, in particular by removing markers that were not genotyped in at least 15 trees per site/local habitat combination. The complete method of AFLP scoring is available in the S1 Method; AFLPs data (binary) are accessible on Dryad (http://dx.doi.org/10.5061/dryad.b2q88).

Genetic structure and spatial genetic structure analysis (SGS)

A Bayesian clustering analysis was performed using STRUCTURE v2.3.4 [56] at both regional and local scale. The analyses were performed with the 'admixture model' and 'correlated allelic frequencies' settings. A burn-in of 10,000 iterations was followed by 100,000 iterations. As we had no *a priori* expectation about the number of clusters to be inferred, the model was run with K (number of clusters) values from K = 1 to K = 10 (five runs were performed for each K value). Trends in L(K) were analyzed using R software, in accordance with the ad-hoc ΔK

method proposed by Evanno, Regnaut and Goudet [57]. STRUCTURE results were summarized using CLUMPAK server [58] to obtain the probability of each individual to belong to each cluster.

Spatial genetic structuring and gene dispersal were assessed on AFLP data using the spatial autocorrelation method based on kinship coefficients, as developed by Hardy and Vekemans [59] and implemented in SPAGeDi v1.3 [60]. Within each site, spatial autocorrelation of kinship coefficient (F_{ij}) was analyzed over twenty evenly spaced distance classes between 0 and 500 m. 95% *null* confidence intervals were obtained through 1000 random permutations of individuals among geographical locations. Neighborhood size (N_b) and gene dispersal (σ_g) were estimated with prior knowledge about population densities, and the slope (b) of the regression of kinship relatedness (F_{ij}) against geographic distance (d_{ij}) was computed with standard error estimated by jack-knifing over loci. SGS intensity was measured as $S_p = b/(F_{(1)}-1)$ where $F_{(1)}$ is the average kinship coefficient between individuals separated by distances belonging to the first distance class.

Landscape-scale analysis of genome-wide divergence

A landscape approach was used to test whether environmental variations were involved in genome-wide genetic divergence. The simultaneous effects of neutral and adaptive sources of genetic divergence were explored through a linear model. More precisely, the model aimed at distinguishing the relative influence of geographic and environmental distances on genetic distance between individuals. Neutral components were estimated both at the regional (based on the membership of individuals relative to different sites) and local scales (based on individual coordinates in a two-dimensional x,y-plane and along a one-dimensional elevation gradient). Adaptive components were modelled through the environmental distance between individuals (soil type, waterlogging frequency and seasonal drought strength). Because light and soil temperature were poorly variable among sites and local habitats, these two factors were excluded from the model (Fig. 2 and S1 Table).

$$\begin{aligned} GENET_{i1,i2} &= \mu + (\theta_1 \times SITE_{i1,i2}) + (1 - SITE_{i1,i2})\left[(\theta_2 \times GEO_{i1,i2}) + (\theta_3 \times ELEV_{i1,i2})\right] \\ &+ (\theta_4 \times DROUGHT_{i1,i2}) + (\theta_5 \times WATERLOG_{i1,i2}) + (\theta_6 \times SOIL_{i1,i2}) + \sigma^2_R \end{aligned}$$

Where $GENET_{i1,i2}$ is the genetic distance between the individuals i1 and i2 (Jaccard distance), μ is the global mean, and σ^2_R the residual variance. SITE $_{i1,i2}$ describes whether the individuals i1 and i2 are from the same site or not (0 = same site, 1 = different sites), $GEO_{i1,i2}$ and $ELEV_{i1,i2}$ are the geographic (Euclidean) distances between individuals inhabiting the same site according to their two-dimensional coordinates in the x,y-plane and their one-dimensional coordinates along an elevation gradient respectively. $DROUGHT_{i1,i2}$, $WATERLOG_{i1,i2}$, and $SOILTYPE_{i1,i2}$ describe the environmental distances between individuals for seasonal drought severity, waterlogging frequency and soil type, according to sites and local habitat environmental properties as described in Table 1. The model was empirically calibrated through a Bayesian method implemented in OpenBUGS [61, 62] (http://www.openbugs.net): 10,000 iterations with a burning of 1,000. A complete description of the model and the BUGS code are provided in S2 Method.

Allele frequency inference

Because properly estimating genotypic frequencies from dominant markers requires prior knowledge of inbreeding coefficient, F_{IS} was estimated from an already published dataset composed of SNPs detected in sequenced ESTs [8] with ARLEQUIN v3.5.1.2 [63]. The mean F_{IS}

(across loci) varied from -0.207 to -0.089 depending on the population considered. Allele frequencies within each study site and local habitat were inferred from AFLPs data based on a mean inbreeding coefficient of -0.14, by solving the standard equation relating inbreeding coefficient, allele frequencies and recessive genotype frequencies for each marker j, with $f(00)$ the relative frequency of the genotype (00) and p the relative frequency of the '0' allele:

$$f(00)_j = (1 - F_{IS})p_j^2 + (F_{IS}p_j),$$

Solving for p:

$$p_j = \frac{-F_{IS} + \sqrt{\Delta_j}}{2(1 - F_{IS})}$$

with

$$\Delta_j = F_{IS}{}^2 - \left[4(1 - F_{IS})\left(-f(00)_j\right)\right]$$

Absolute frequencies were obtained by multiplying relative frequencies by twice the sample size in each subpopulation, rounding to the nearest integer. These absolute frequencies were used in all subsequent analyses of population differentiation and outlier detection.

Intra-site differentiation

For each study site, locus-specific genetic differentiation (F_{ST}) between local habitats was estimated from inferred genotypic data through a classical analysis of molecular variance (AMOVA [64]) using ARLEQUIN v3.5.1.2 (Slatkin's method).

Detection of outlier loci

Excess divergence within populations inhabiting contrasting habitats was tested based on two F_{ST}-based approaches:

a. the coalescent-based FDIST method [65] implemented in ARLEQUIN v3.5.1.2 [63]. We implemented both a hierarchical island model including the two study sites simultaneously, plus two classical island models for each site separately (within-Laussat and within-Régina respectively). False-discovery rate was assessed according to Strimmer's method [66, 67]: p-values obtained from the coalescent method were converted into q-values using the '*fdrtool*' package in R [67], and the latter was used to set an FDR threshold of 0.10.

b. the Bayesian method implemented in BAYESCAN [34], with an FDR threshold of 0.10.

For each outlier detected, X^2 tests were performed on AFLP band frequencies to test the hypothesis of equal frequencies between local habitats within each study site.

Evaluating Type I and Type II error rates

Both the Bayesian- and the coalescent-based methods were submitted to a sensitivity analysis by estimating Type I and Type II error rates. To do this, we simulated one-hundred datasets with the same sample size and number of markers as our empirical datasets (two groups of two populations with divergence between groups F_{CT} = 0.01). Out of the 1196 simulated markers, 1146 were simulated with average F_{ST} = 0.039 and F_{ST} = 0.026 (equal to empirical within-site F_{ST} values, corresponding to α = 0 in the Bayesian framework), 25 were constrained at F_{ST} = 0.11 (Bayesian α = 3) and 25 at F_{ST} = 0.23 (Bayesian α = 5) to simulate zero, moderate and strong selection respectively. The simulations were submitted to the same outlier detection

analyses as the empirical dataset, and the average number of significant markers in each class, over the global set of one-hundred simulations, were reported. The ratio of number of neutral markers detected as significant, over the total number of neutral markers, was taken as an estimate of Type I error rate. The number of markers under selection not detected as significant, out of the total number of markers under selection, was taken as an estimate of Type II error rate.

Results

AFLP data

After data cleaning, 53.3% of markers (corresponding to 1196 bins out of 2242) were retained for further analysis as described in S1 Method. The binset is available on Dryad (http://dx.doi.org/10.5061/dryad.b2q88).

Blind analysis of population structure

L(K) was high from $K = 1$ to $K = 7$ for the regional-scale analysis (S2 Fig.), from $K = 1$to $K = 7$ within Laussat, and from $K = 1$to $K = 5$ within Regina (S3 and S4 Figs.). At the regional level, a maximum peak of ΔK was detected at $K = 3$: individuals from 'Régina' were assigned to one cluster, while the individuals from 'Laussat' were assigned to two clusters concordant with local habitats (S2 Fig.). At $K = 2$, the inferred clusters distinguished the trees inhabiting the two study sites of Laussat and Regina. At intra-site level, a maximum peak of ΔK was detected at $K = 2$ in Laussat, and at $K = 5$ in Régina (S3 and S4 Figs.). In Laussat, the genetic clusters inferred at $K = 2$ were geographically grouped in agreement with local habitat patchiness (S3 Fig.), except for five trees of hilltop assigned to the same genetic clusters than the trees inhabiting the bottomland. In Régina the genetic structure was not clear at $K = 5$ as the individuals were assigned to the different clusters with quasi-equal probabilities, indicating a complete admixture and the probable absence of genetic structuring (S4 Fig.).

Spatial Genetic Structure and gene dispersal within populations

Spatial genetic structure (SGS) was assessed by estimating relative relatedness in 1711 pairs of individuals in Régina and 1810 pairs in Laussat. The mean number of pairs by distance class was 86 in Laussat and 92 in Régina. Significant SGS were detected in both sites (Fig. 3 and Table 2), with kinship declining with increasing geographical distance (b = -0.016 ±0.001 in Laussat and b = -0.012 ±0.001 in Regina). In Laussat, spatial autocorrelation was significantly positive until 56 m, and became significantly negative from 230 m onward, with a neighborhood size (N_b) of 65.6. In Régina, autocorrelation was positive and significant until 30 m and became negative and significant beyond 250 m, with a neighborhood size $N_b = 78.5$. In both sites, the autocorrelation was positive at a distance corresponding to the distance separating trees inhabiting the same habitat, and became negative at a distance corresponding to the distance separating trees inhabiting two distinct habitats. Gene dispersal was estimated at 45.7 and 64.4 m in Laussat and Régina respectively. We also checked that spatial genetic structure did not vary among local habitats: significant SGS were detected in both habitats until 20 and 30 m in Laussat, and until 20 m in Régina (S5 Fig.), and we did not detect differences in SGS among local habitat types within sites, on the basis of the extent of relatedness ($F_{i,j}$), SGS intensity (S_p) and slope (b), S5 Fig.

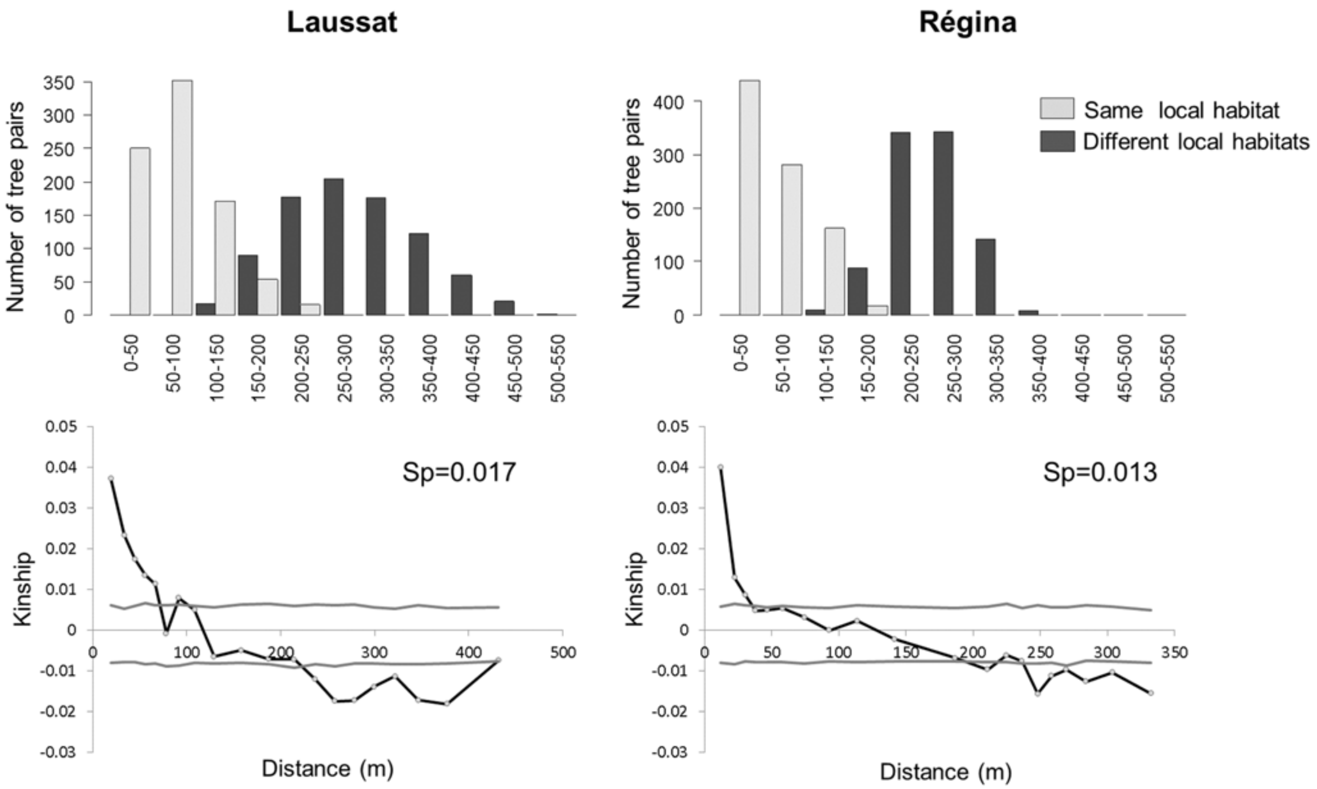

Fig 3. Top: Number of tree pairs in each distance class. Bottom: Intra-site spatial genetic structure (SGS) analysis based on all AFLP markers.

doi:10.1371/journal.pone.0121394.g003

Landscape scale analysis of genetic divergence

Partitioning the genetic distance into neutral and adaptive processes through a landscape Bayesian model revealed a strong 'site' effect on the genetic distance between individuals ($\theta 1 = 1.71.10^{-2}$, Table 3 and Fig. 4). Within site, we detected a positive relationship between the geographic distance in the two-dimensional x,y-plane and the genetic distance between individuals: $\theta 2 = 4.2.10^{-5}$ m^{-1} (i.e. the mean genetic distance between individuals increases of 0.042 every kilometer). However, there was no positive trend elevation and genetic distances. Among environmental sources of genetic divergence, waterlogging frequency was positively related with genetic distance ($\theta 5 = 1.5.10^{-2}$).

Table 2. SGS and gene dispersal parameters estimated by SpaGeDi.

	Parameter	Laussat	Regina
**SGS parameter estimates**	b (SE)	-0.016 (0.001)	-0.012 (0.001)
	F1 (SE)	0.037 (0.003)	0.04 (0.032)
	Sp	0.017	0.013
**Gene dispersal parameter estimates**	D	0.005	0.003
	Nb (SE)	65.62 (21.02)	78.51 (14.21)
	σg (SE)	45.7 (7.33)	64.4 (5.82)

*F*1 is the autocorrelation of kinship coefficient in the first distance class, b is the slope of the regression between relatedness (F_{ij}) and geographic distance (d_{ij}), *Sp* is SGS intensity, D is population density, Nb is Neighborhood size, and σg is gene flow estimate.

doi:10.1371/journal.pone.0121394.t002

Table 3. Parameters inferred by the landscape Bayesian model with their respective posterior probabilities (mean, standard deviation, median, and 95% credible interval): μ (global mean), θ_1 (site effect), θ_2 (slope of the relation between geographical and genetic distance within sites according to a 2D x,y-plane), θ_3 (slope of the relation between the geographical and genetic distance within sites according to an elevation gradient), θ_4 (drought severity effect), θ_5 (waterlogging frequency effect), and θ_6 (soil type effect).

	mean	sd	val2.5pc	median	val97.5pc
μ	1.95×10^{-01}	6.82×10^{-04}	1.93×10^{-01}	1.95×10^{-01}	1.96×10^{-01}
θ1	1.70×10^{-02}	1.03×10^{-03}	1.52×10^{-02}	1.71×10^{-02}	1.92×10^{-02}
θ2	4.19×10^{-05}	5.81×10^{-06}	3.05×10^{-05}	4.19×10^{-05}	5.32×10^{-05}
θ3	-1.85×10^{-04}	5.86×10^{-05}	-2.98×10^{-04}	-1.85×10^{-04}	-6.93×10^{-05}
θ4	-2.57×10^{-03}	6.59×10^{-04}	-3.88×10^{-03}	-2.56×10^{-03}	-1.29×10^{-03}
θ5	1.45×10^{-02}	8.54×10^{-04}	1.28×10^{-02}	1.45×10^{-02}	1.62×10^{-02}
θ6	-1.02×10^{-02}	9.91×10^{-04}	-1.22×10^{-02}	-1.02×10^{-02}	-8.23×10^{-02}

doi:10.1371/journal.pone.0121394.t003

Genetic differentiation among subpopulations inhabiting contrasted habitats and outlier detection

Overall Slatkin's F_{ST} between local habitats was respectively 0.03 (sd = 0.07) in Laussat and 0.02 (sd = 0.05) in Régina (S6 Fig.). Locus-specific F_{ST} was significant for 8.1% and 6.1% of loci in Laussat and Régina respectively. Under the hierarchical island model, the extent of differentiation between study sites was F_{CT} = 0.01 (sd = 0.05), while the differentiation between local habitats within sites was F_{SC} = 0.03 (sd = 0.05) and the differentiation between local habitats among sites F_{ST} = 0.04 (sd = 0.06), Fig. 5.

After local false-discovery rate assessment [66, 67], 42 loci were detected as outliers being under divergent selection in at least one analysis (under a FDR threshold of 10%), Table 4. Under the hierarchical coalescent model, fifteen (1.25%) outlier loci were detected between subpopulations within regions (F_{ST}), Fig. 6 and Table 4 (column 2). The within-site coalescent analyses revealed fifteen (1.35%) and eighteen (1.65%) outliers respectively for Laussat and Régina (out of 1109 and 1090 polymorphic markers respectively), Table 4 (columns 6 and 11). Among all outliers detected by the hierarchical model, four were also detected by the within-site coalescent model in Laussat (loci 345, 485, 624 and 742), and one in Régina (locus 463). Locus 46 was detected by both within-site analyses but not in the hierarchical model. The

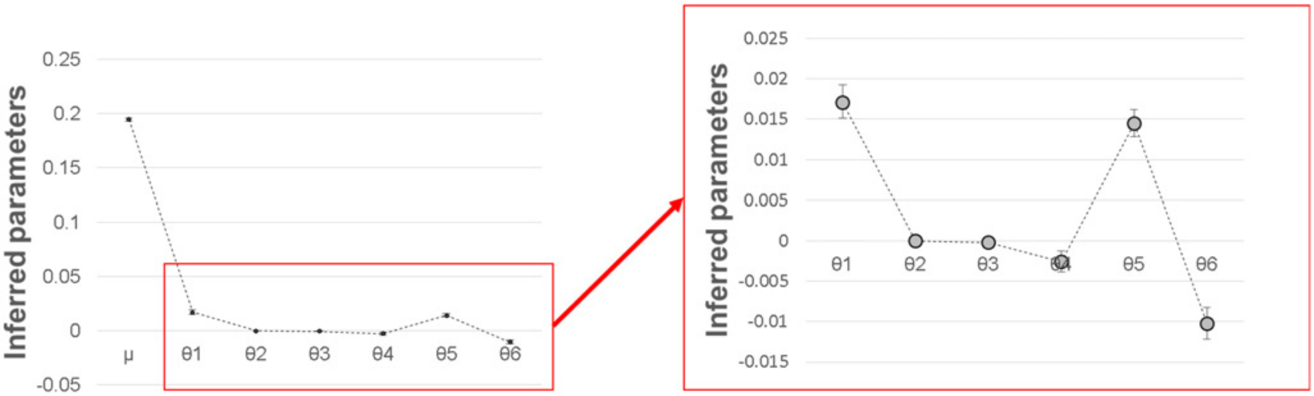

Fig 4. Results of the landscape-scale Bayesian model (values are provided in Table 3). Points show the inferred parameters with their 95% posterior probability: μ is the global mean, θ_1 represents the effect of site, θ_2 is the slope of the relation between the geographical and genetic distance within sites in a two-dimensional x,y-plane, θ_3 is the slope of the relation between the geographical and genetic distance within sites according to an elevation gradient, θ_4 describes the effect of drought severity, θ_5 describes the effect of waterlogging frequency and θ_6 describes the effect of soil type.

doi:10.1371/journal.pone.0121394.g004

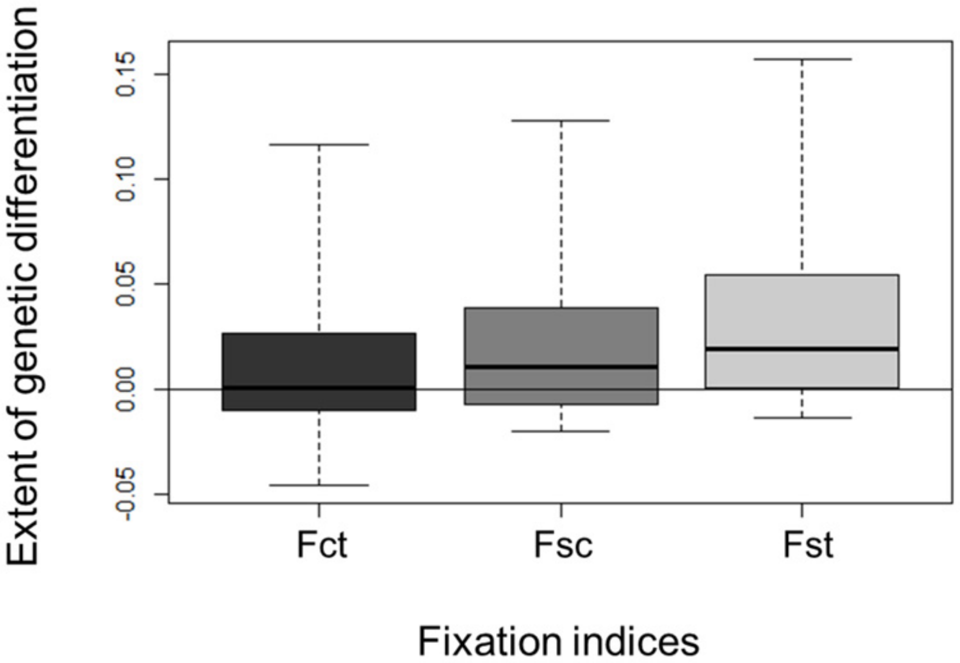

Fig 5. Box-plot of the distribution of single-locus fixation indices (F_{CT}, F_{SC} and F_{ST}) estimated under the hierarchical model of population subdivision (boxes indicate 5%, 25%, 50%, 75% and 95% quantiles). F_{CT} is the differentiation between sites relative to total, F_{SC} is the differentiation between local habitats relative to sites, and F_{ST} is the differentiation between local habitats relative to total.

doi:10.1371/journal.pone.0121394.g005

Bayesian analysis detected four outliers (loci 86, 345, 485 and 624, FDR = 0.084 and FNDR = 0.092) in Laussat, and two (loci 313 and 962, FDR = 0.01 and FNDR = 0.091) in Regina (Fig. 7 and Table 4, columns 7 and 12). All outliers detected by the Bayesian methods were also detected by within-site coalescent analyses (loci 86, 345, 485 and 624 in Laussat, loci 313 and 962 in Régina), and three (loci 345, 485 and 624) by the hierarchical model as well. Simulations were used to assess Type I and Type II error rates. For the coalescent method, Type I error rate was $\alpha = 0.3\%$ and Type II error rate was $\beta = 57\%$ for both sites; error rates were similar for the Bayesian method ($\alpha = 0.3\%$ and $\alpha = 0.2\%$ for Laussat and Régina respectively; $\beta = 54\%$ for both sites).

To check whether variations in AFLP band frequencies between local habitats were consistent with the hypothesis of selection acting in the same direction in the two replicates, we compared the direction of inter-habitat variation in band frequencies between the two study sites for outlier loci, Fig. 8. About half of all detected outliers showed the same trend of frequency variations in the two study sites. For loci 30, 233, 416, 624, 668, 785, 791, 871 and 955, the frequency of '1' (band presence) was higher in hilltop than in bottomland in both sites. For loci 19, 46, 54, 221, 313, 359,468, 485, 757, and 860, the frequency of '1' was higher in bottomland than in hilltop in both sites. However, X^2 tests revealed significant differences in AFLP band frequency between local habitats in at least one study site for only fifteen outliers (46, 54, 221, 233, 313, 359, 468, 485, 624, 757, 787, 791, 860, 871 and 955). Finally, congruent patterns of AFLP band divergence between local habitats were supported by significant X^2 tests in the two study sites for only three outliers: 46, 757 and 871. Six loci were monomorphic in one study site: loci 69, 345, 451, 463, 799 and 848.

Table 4. Summary of outliers detected in at least one analysis.

Locus	Hierarchical approach	Within-Laussat classical approach (1109 polymorphic markers)					Within-Regina classical approach (1090 polymorphic markers)				
	Coalescent outlier detection under a hierarchical model (1196 polymorphic markers)	Slatkin FST within Laussat	Fst Pval	X^2 test on AFLP band frequency within Laussat (p-value)	Coalescent outlier detection under a classical island model within Laussat	Bayesian outlier detection within Laussat	Slatkin FST within Regina	Fst Pval	X^2 test on AFLP band frequency within Régina (p-value)	Coalescent outlier detection under a classical island model within Régina	Bayesian outlier detection within Régina
19	ns	-0.014	0.454	0.6797	ns	ns	0.238	0.002	0.1094	*	ns
30	ns	0.184	0.001	0.1084	ns	ns	0.229	0.001	0.09	*	ns
41	ns	-0.029	1.000	1	Ns	ns	0.177	0.009	0.005*	*	ns
46	ns	0.290	0.000	0.036*	*	ns	0.311	0.000	0.0295*	*	ns
54	ns	0.148	0.011	0.072	ns	ns	0.336	0.000	0.0025*	*	ns
69	ns	-0.021	1.000	Na	ns	ns	0.304	0.000	0.1014	*	ns
86	ns	0.435	0.000	0.002*	*	*	-0.032	1.000	1	ns	ns
158	*	-0.020	0.801	0.7026	ns	ns	-0.017	0.718	0.7156	ns	ns
181	*	0.078	0.025	0.054	ns	ns	0.014	0.268	0.2649	ns	ns
221	ns	0.000	0.443	0.4123	ns	ns	0.339	0.000	0.009*	*	ns
233	*	0.002	0.458	0.4873	ns	ns	0.090	0.019	0.0395*	ns	ns
288	ns	0.268	0.000	0.045*	*	ns	0.046	0.160	1	ns	ns
291	ns	-0.024	1.000	1	ns	ns	0.218	0.000	0.0375*	*	ns
313	ns	0.042	0.114	0.2854	ns	ns	0.417	0.000	0.0005*	*	*
345	*	0.383	0.000	0.0005*	*	*	na	na	na	na	na
359	ns	-0.005	0.430	0.5557	ns	ns	0.231	0.000	0.0005*	*	ns
416	ns	0.014	0.187	0.6192	ns	ns	0.222	0.001	0.0915	*	ns
451	*	0.200	0.000	0.002*	ns	ns	na	na	na	na	na
457	ns	0.350	0.000	0.006*	*	ns	-0.007	0.478	0.5797	ns	ns
463	*	na	na	na	na	na	0.265	0.000	0.0005*	*	ns
468	ns	0.334	0.000	0.0115*	*	ns	0.135	0.006	0.0595	ns	ns
485	*	0.548	0.000	0.0005*	*	*	-0.024	0.771	0.7251	ns	ns
585	ns	0.334	0.000	0.011*	*	ns	0.114	0.009	0.4838	ns	ns
605	*	0.017	0.500	0.4848	ns	ns	-0.018	0.833	1	ns	ns
624	*	0.638	0.000	0.0005*	*	*	0.129	0.010	0.2009	ns	ns
668	*	0.006	0.295	0.2779	ns	ns	-0.014	1.000	1	ns	ns
715	ns	-0.024	0.811	1	ns	ns	0.235	0.001	0.0895	*	ns
742	*	0.315	0.000	0.0005*	*	ns	-0.018	1.000	1	ns	ns
743	ns	0.329	0.001	0.001*	*	ns	-0.031	1.000	1	ns	ns
748	ns	0.129	0.005	0.0865*	ns	ns	0.290	0.000	0.009*	*	ns
757	ns	0.307	0.000	0.001*	*	ns	0.089	0.033	0.0465*	ns	ns
785	*	0.239	0.001	0.005*	ns	ns	0.066	0.096	0.1434	ns	ns
791	ns	0.035	0.100	0.3653	ns	ns	0.294	0.000	0.035*	*	ns
799	*	-0.023	1.000	na	ns	ns	0.002	0.311	0.4253	ns	ns
848	*	0.195	0.002	0.0405*	ns	ns	-0.022	1.000	na	ns	ns
860	ns	0.388	0.000	0.0005*	*	ns	0.095	0.009	0.0425*	ns	ns
868	ns	0.354	0.000	0.006*	*	ns	0.020	0.236	0.4113	ns	ns

(*Continued*)

Table 4. (*Continued*)

Locus	Hierarchical approach	Within-Laussat classical approach (1109 polymorphic markers)					Within-Regina classical approach (1090 polymorphic markers)				
	Coalescent outlier detection under a hierarchical model (1196 polymorphic markers)	Slatkin FST within Laussat	Fst Pval	X^2 test on AFLP band frequency within Laussat (p-value)	Coalescent outlier detection under a classical island model within Laussat	Bayesian outlier detection within Laussat	Slatkin FST within Regina	Fst Pval	X^2 test on AFLP band frequency within Régina (p-value)	Coalescent outlier detection under a classical island model within Régina	Bayesian outlier detection within Régina
871	ns	0.209	0.000	0.001*	ns	ns	0.255	0.000	0.0005*	*	ns
874	ns	-0.016	1.000	1	ns	ns	0.223	0.000	0.0005*	*	ns
881	*	0.226	0.001	0.007*	ns	ns	0.002	0.319	0.3113	ns	ns
955	ns	0.301	0.002	0.0355*	*	ns	0.006	0.334	0.3533	ns	ns
962	ns	-0.017	1.000	1	ns	ns	0.257	0.000	0.0005*	*	*

*: significant

ns: non-significant

na: missing value

doi:10.1371/journal.pone.0121394.t004

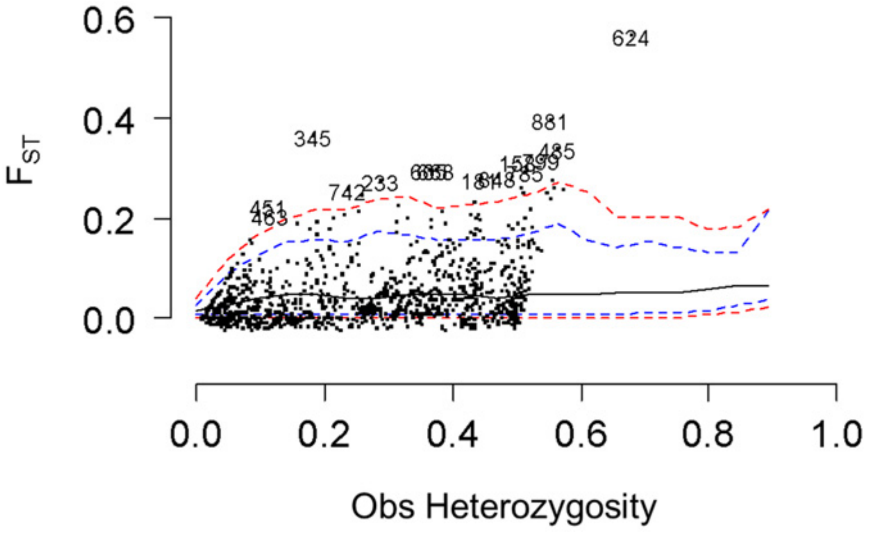

Fig 6. Results of the coalescent outlier search under the hierarchical island model. Blue dashed line: 95% neutral envelop; red dashed line: 99% neutral envelop. Only loci above the neutral envelop and retained after multiple corrections (FDR = 10%) are shown.

doi:10.1371/journal.pone.0121394.g006

Discussion

The genetic clusters inferred by STRUCTURE were spatially aggregated. At regional scale, the genetic structuring among populations inhabiting different study sites ($K = 2$) can easily be explained by isolation-by-distance. To evaluate the role of neutral processes in shaping within-population genetic structure, we investigated the fine-scale genetic structuring over all loci within each study site. Kinship coefficients decreased with geographical distances in the two study sites as expected under the isolation-by-distance model. Gene flow estimates were very low in both sites (around 50 m), and the lower gene flow estimated in Laussat (45.7 m against

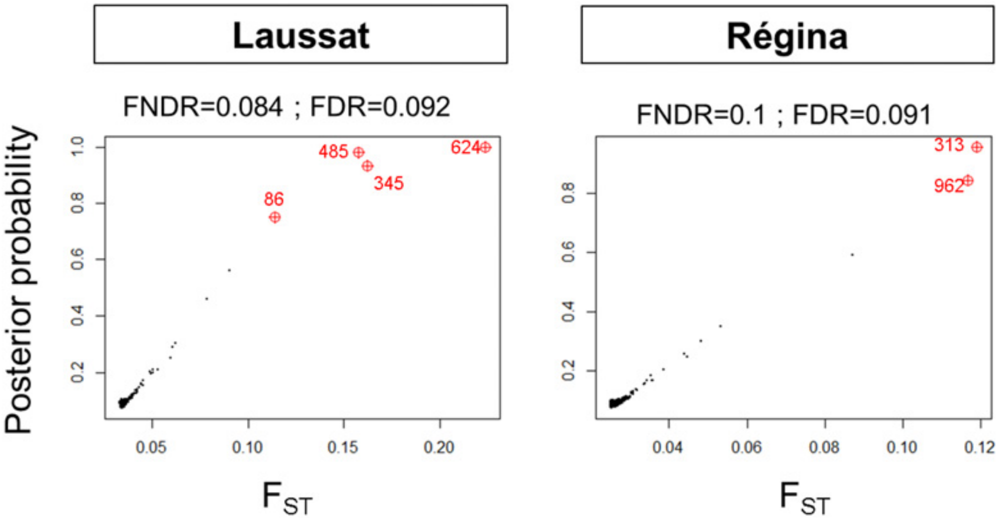

Fig 7. Results of the Bayesian outlier search under a 10% expected FRD.

doi:10.1371/journal.pone.0121394.g007

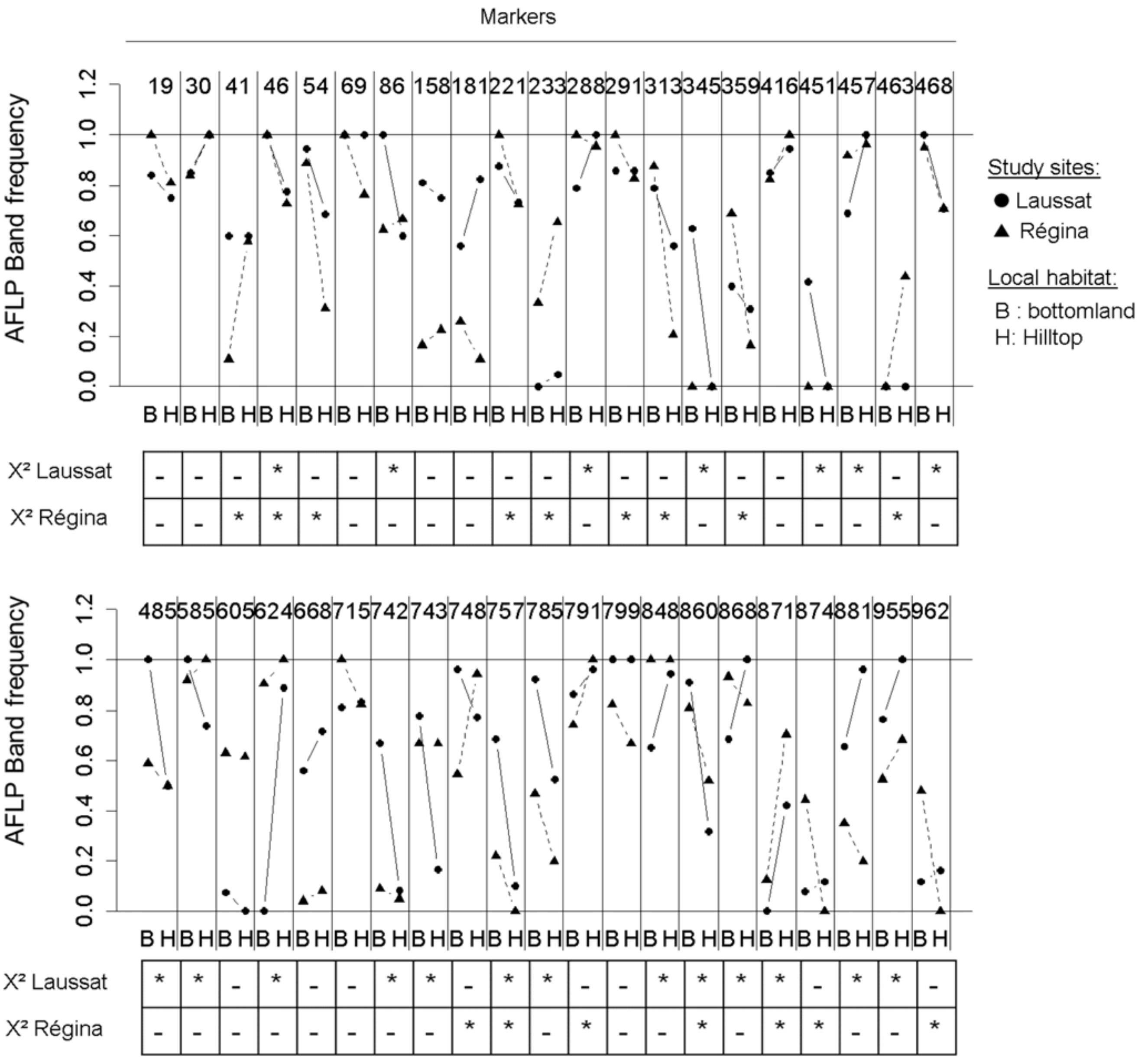

Fig 8. Band presence frequency (allele '1') in each local habitats ('B': bottomland, 'H': hilltop) and each study site (circles: Laussat, squares: Régina). The tables below show the result of X² tests on AFLP band frequency ('*': significant; '-': non-significant or missing).

doi:10.1371/journal.pone.0121394.g008

64.4 m in Régina) was concordant with the stronger genome-wide genetic structuring among local habitats in that site. Similar SGS patterns have been observed in many temperate [14, 39, 42, 68–72] and tropical tree species [73–79] (including the Guiana shield [13, 20, 80–82]), and they are likely to be caused by neutral processes (restrictions in gene flow and local inbreeding [80]). In tropical trees, pollen dispersal is commonly restricted to short distances, and seed dispersal is often highly restricted in autochorous species [13, 16, 22] causing the clumping of maternal progeny groups. Consequently, mating among neighboring relatives—which may be frequent in dense populations—commonly results in local inbreeding. In autochorous *E.*

falcata, gene dispersal estimates (σ_g ranging from 45.7 to 64.4 m depending on the study site) are in agreement with these characteristics [71, 83].

The slight variations in the fine-scale spatial genetic structuring between study sites and between local habitats within sites may possibly be caused by variations in environmental conditions and their direct effect on pollen and seed dispersal. For example, fine-scale spatial genetic structure and population differentiation were weaker, and gene flow was slightly higher, in the study site of Régina (where the relief is steep with abrupt slopes and precipitation more abundant with about 3500 mm/year) than in Laussat (where the relief is quite flat and precipitation does not exceed 2500 mm/year). Variations in topography (relief and slopes), rainfall and water flows may have a direct effect on gene flow and SGS. Even if the spatial genetic structuring was quite similar among habitat types, it was slightly weaker in the bottomland than in the plateau in Laussat, possibly because water flows caused by intense waterlogging contribute to scatter seeds and to increase gene flow in this habitat.

The existence of genome-wide neutral divergence directly caused by geographic distances (between and within study sites) was also corroborated by the landscape-scale analysis of genetic divergence. Indeed, the inferred parameter θ1 revealed a site effect on the genetic divergence. Even if it is more likely to be caused by neutral processes related to the geographic distance itself (isolation-by-distance), this parameter may also capture genome-wide adaptive processes caused by variations in both abiotic and biotic conditions between the study sites (e.g. variation in rainfall, population density, competition levels, etc.). The parameter θ2 captured the effect of geographic distances on genetic distances within study sites (in the two-dimensional x,y-plane), confirming the existence of a neutral spatial genetic structuring within study sites.

In addition to neutral, distance-based, genome-wide divergence, variations in waterlogging frequency may constitute a source of adaptive genetic divergence as revealed by a positive estimate of the parameter θ5. Surprisingly, the waterlogging effect was of the same order of magnitude as the site effect, despite the large differences in geographical scales separating sites (hundreds of kilometers) and local habitats (hundreds of meters). This implies that a fraction of genome-wide divergence may have been caused by 'pervasive selection' [8, 69] over microgeograhical scales, as expected under the isolation-by-adaptation (IBA) model [84]. Indeed, indirect estimates of gene flow in well-established adult populations represent the 'effective' gene flow. They do not depend on seed and pollen dispersal only, but also on the ability of seedlings to establish and grow in the environment where they were dispersed (i.e. on local adaptation processes), that is particularly true for immobile organisms. The genetic differentiation between local subpopulations (F_{ST} = 0.04; sd = 0.06) was large regarding the differentiation between sites (F_{CT} = 0.01; sd = 0.05), despite the geographical scales involved (about 300 km among sites, up to 200 m between local habitats). As the effects of dispersal limitation can only increase with distance, it seems unlikely that this kind of process would be stronger locally than at the regional level. This means that genome-wide divergence may be influenced by local adaptation to micro-environmental variability. In particular, waterlogging frequency influence genome-wide divergence over microgeographical scales as revealed by the landscape-scale approach, probably through its direct effect on seedlings establishment.

Locus-specific footprints of local adaptation were also detected for a fraction of the analyzed loci. Indeed, adaptive divergence may either affect many genes of low effects, or a reduced number of targeted loci (few genes of major effects) involved in key metabolic or physiologic pathways, themselves involved in fitness [85–92]. Both the coalescent and the Bayesian method allowed the detection of outliers at the microgeographical scale. Among the 42 (3.5%) outliers detected with the coalescent method, 6 were validated by the Bayesian method (0.5%, loci 85, 313, 345, 485, 624, 962) and are strong candidate targets of divergent selection [34, 65, 93].

Precision tests performed through simulations showed that false-positive outliers should be very rare (a fraction of a percent) making it unlikely that a large fraction of the detected outliers are artifacts. Nineteen interesting outliers showed similar trends of band frequency variations between local habitats in the two study sites: twelve were supported by significant variations in AFLPs band frequency among local habitats (X^2 test) in one study site, and three were supported by significant variations in AFLPs band frequency among local habitats in the two study sites. This result indicates that these outliers may be true positives and that divergent selection would have driven variations in genotypic frequencies among local habitats in the same direction in the two study sites. The majority of outliers were, however, detected in only one study site. This can be ascribe to a lack of statistical power and/or to environmental differences between the study sites. Indeed, simulations revealed that 50% of loci undergoing moderate to strong selection would go undetected. Alternatively, different selective pressures caused by different selective agents may be involved in the adaptive genetic divergence within the two study sites. Moreover, even assuming that the same selective agents occur in the two sites, different multi-locus combinations of alleles or different loci may have been selected in the two populations. In the case of traits under multi-genic control, it is hard to detect single targets of selection of low strength and to identify conserved single-locus divergence patterns [94].

Outliers may also indicate the presence of some other indirect mechanisms inducing genetic divergence that may not be directly related to environmental filters [65, 95, 96]. Outlier tests based on a differentiation index (F_{ST}) are robust to inter-locus variations, and theoretical models show that footprints of natural selection persist longer in differentiation indices (F_{ST}) than in intra-population estimators of genetic diversity [36]. F_{ST}-based methods are also supposed to be robust to many demographic scenarios [97, 98], partly because demographic events affect the genome in a homogeneous manner [89]. However, the inclusion of bottlenecked populations may bias the method [36]. Even if trees were sampled in mature and supposedly undisturbed forests, we have no evidence that the studied populations have not experienced a recent demographic change (bottleneck or expansion), and the degree to which these tests are robust to demography has not yet been fully explored [99].

Scans for outlier detection are abundant in the literature for a variety of geographical scales and biological models, including animals and plants, both aquatic and terrestrial [8, 84, 91, 97, 100–107]. The proportion of outliers for selection detected was low, but surprisingly high when considering the microgeographical scale studied here. This suggests that the same processes that occur with a larger degree of spatial separation in other species may occur at very short distances in *E. falcata*. These loci may be involved in metabolic pathways crucial for seedlings establishment and growth under the particular constraints imposed by each habitat, such as waterlogging and hypoxia experienced in bottomlands, or seasonal soil drought experienced in plateaus. However, the selective agency behind the observed divergence needs to be functionally proven by showing that the putatively selected polymorphisms control adaptive traits. This will require (i) identifying the genes involved in fitness-related phenotypic traits, and (ii) targeting these loci for testing local adaptation on candidate genes [108].

Nevertheless, the patterns of divergence observed in this study are in agreement with previous reports based on SNPs within ESTs [8] and quantitative phenotypic traits [30], and reinforce the idea that adaptive phenomena may affect a substantial fraction of the genome at microgeographical scales in Neotropical tree populations. The example provided by *E. falcata* is a piece of evidence that evolution may drive genetic differentiation and subpopulation divergence even in conditions in which gene flow may easily erase the effects of weak selective forces (i.e. over microgeographical scales in continuous stands of high population densities with extensive gene flow). At such spatial scales, dispersal and population connectivity (which are the field of landscape genetics [109]) meet evolutionary processes (which are the field of

population and ecological genetics) providing a deeper understanding of the ecological processes responsible for the maintenance of biodiversity. Indeed, adaptive divergence caused by microgeographic habitat patchiness may constitute the fuel that feeds the great diversity harbored by the tropical rainforest of Amazonia. The genetic diversity of wild populations in turn conditions their adaptive potential (i.e. their ability to adapt to environmental variations) and consequently their ability to persist when undergoing environmental changes. The present study suggests that understanding evolutionary processes in tropical rainforests and in plant populations more widely should require particular attention on microgeographic divergence and local adaptation.

Supporting Information

S1 Fig. Environmental characterization: Canopy opening and pedology. A: Example of hemispherical photograph done in the plateau of Laussat, B and C: Examples of soil toposequences (B: hygromorphic soil of Laussat bottomland, C: ferralitic soil of Laussat plateau).
(TIF)

S2 Fig. Bayesian clustering analysis on the whole data set. Upper pane: L(K) and ΔK values. Middle pane: individual α values for $K = 2$ and $K = 3$. Lower pane: geographical distribution of individuals belonging to the main clusters (see text).
(TIF)

S3 Fig. Bayesian clustering analysis on the Laussat data set. Upper pane: L(K) and ΔK values. Middle pane: individual α values for $K = 2$. Lower pane: geographical distribution of individuals belonging to the main clusters (see text).
(TIF)

S4 Fig. Bayesian clustering analysis on the Régina data set. Upper pane: L(K) and ΔK values. Middle pane: individual α values for $K = 5$. Lower pane: geographical distribution of individuals belonging to the main clusters (see text).
(TIF)

S5 Fig. Intra-habitat spatial genetic structure analysis based on all AFLP markers.
(TIF)

S6 Fig. Density distribution of Slatkin's locus-specific F_{ST} overall loci and for loci displaying a significant F_{ST}.
(TIF)

S1 Method. AFLP scoring.
(DOCX)

S2 Method. Model description and BUGS Code.
(DOCX)

S1 Table. Environmental conditions in each of the study sites and local habitat.
(DOCX)

Acknowledgments

We thank Saint-Omer Cazal and Julien Engel for technical assistance, and Bruno Ferry for soil characterization. We also thank météo-FRANCE for rainfall/ETP data for the stations of 'Iracoubo' and 'Régina'.

Author Contributions

Analyzed the data: LB. Wrote the paper: LB IS CSS MF. Conceived the experiment: LB IS. Designed the experiment: LB. Performed the experiment: LB. Helped perform the experiment: CSS.

References

1. Nevo E, Beiles A, Storch N, Doll H, Andersen B. Microgeographic edaphic differentiation in hordein polymorphisms of wild barley. Theor Appl Genet. 1983; 64: 123–132. doi: 10.1007/BF00272719 PMID: 24264871
2. Richardson JL, Urban MC, Bolnick DI, Skelly DK. Microgeographic adaptation and the spatial scale of evolution. Trends Ecol Evol. 2014; 29: 165–176. doi: 10.1016/j.tree.2014.01.002 PMID: 24560373
3. Bradshaw A. Population differentiation in *Agrostis tenuis* Sibth. III. Populations in varied environments. New Phytol. 1960; 59: 92–103.
4. Jain SK, Bradshaw AD. Evolutionary divergence among adjacent plant populations. I. The evidence and its theoretical analysis. Heredity. 1966; 21: 407–441.
5. Schmitt J, Gamble SE. The effect of distance from the parental site on offspring performance and inbreeding depression in *Impatiens capensis*: A Test of the local adaptation hypothesis. Evolution. 1990; 44: 2022–2030.
6. Hamrick JL, Allard RW. Microgeographical variation in allozyme frequencies in *Avena barbata*. Proc Natl Acad Sci U S A. 1972; 69: 2100–2104. PMID: 16592002
7. Csilléry K, Lalagüe H, Vendramin GG, González-Martínez SC, Fady B, Oddou-Muratorio S. Detecting short spatial scale local adaptation and epistatic selection in climate-related candidate genes in European beech (*Fagus sylvatica*) populations. Mol Ecol. 2014; 23: 4696–4708. doi: 10.1111/mec.12902 PMID: 25156570
8. Audigeos D, Brousseau L, Traissac S, Scotti-Saintagne C, Scotti I. Molecular divergence in tropical tree populations occupying environmental mosaics. J Evol Biol. 2013; 26: 529–544. doi: 10.1111/jeb.12069 PMID: 23286313
9. Turner TL, Bourne EC, Von Wettberg EJ, Hu TT, Nuzhdin SV. Population resequencing reveals local adaptation of *Arabidopsis lyrata* to serpentine soils. Nat Genet. 2010; 42: 260–263. doi: 10.1038/ng.515 PMID: 20101244
10. Linhart YB, Grant MC. Evolutionary significance of local genetic differentiation in plants. Annu Rev Ecol Syst. 1996; 27: 237–277.
11. Nevo E. Evolution of genome—phenome diversity under environmental stress. Proc Natl Acad Sci U S A. 2001; 98: 6233–6240. PMID: 11371642
12. Jump AS, Penuelas J. Running to stand still: adaptation and the response of plants to rapid climate change. Ecol Lett. 2005; 8: 1010–1020.
13. Hardy OJ, Maggia L, Bandou E, Breyne P, Caron H, Chevalier M-H, et al. Fine-scale genetic structure and gene dispersal inferences in 10 Neotropical tree species. Mol Ecol. 2006; 15: 559–571. PMID: 16448421
14. Vekemans X, Hardy OJ. New insights from fine-scale spatial genetic structure analyses in plant populations. Mol Ecol. 2004; 13: 921–935. PMID: 15012766
15. Degen B, Roubik DW. Effects of animal pollination on pollen dispersal, selfing, and effective population size of tropical trees: a simulation study. Biotropica. 2004; 36: 165–179.
16. Ward M, Dick CW, Gribel R, Lowe AJ. To self, or not to self: A review of outcrossing and pollen-mediated gene flow in neotropical trees. Heredity. 2005; 95: 246–254. PMID: 16094304
17. Petit RJ, Hampe A. Some evolutionary consequences of being a tree. Annu Rev Ecol Evol Syst. 2006; 37: 187–214.
18. Hamrick JL. Response of forest trees to global environmental changes. For Ecol Manage. 2004; 197: 323–335.
19. Bacles CFE, Jump AS. Taking a tree's perspective on forest fragmentation genetics. Trends Plant Sci. 2011; 16: 13–18. doi: 10.1016/j.tplants.2010.10.002 PMID: 21050799
20. Degen B, Bandou E, Caron H. Limited pollen dispersal and biparental inbreeding in *Symphonia globulifera* in French Guiana. Heredity. 2004; 93: 585–591. PMID: 15316558
21. Veron V, Caron H, Degen B. Gene flow and mating system of the tropical tree *Sextonia rubra*. Silvae Genet. 2005; 54: 275–280.

22. Dick C, Hardy O, Jones F, Petit R. Spatial scales of pollen and seed-mediated gene flow in tropical rain forest trees. Trop Plant Biol. 2008; 1: 20–33.

23. Sagnard F, Oddou-Muratorio S, Pichot C, Vendramin G, Fady B. Effects of seed dispersal, adult tree and seedling density on the spatial genetic structure of regeneration at fine temporal and spatial scales. Tree Genet Genomes. 2011; 7: 37–48.

24. Ferry B, Morneau F, Bontemps J-D, Blanc L, Freycon V. Higher treefall rates on slopes and water-logged soils result in lower stand biomass and productivity in a tropical rain forest. J Ecol. 2010; 98: 106–116.

25. Kahn F. The distribution of palms as a function of local topography in Amazonian terra-firme forests. Experientia. 1987; 43: 251–259.

26. Fortunel C, Fine PVA, Baraloto C. Leaf, stem and root tissue strategies across 758 Neotropical tree species. Funct Ecol. 2012; 26: 1153–1161.

27. Kraft NJ, Valencia R, Ackerly DD. Functional traits and niche-based tree community assembly in an amazonian forest. Science. 2008; 322: 580–582. doi: 10.1126/science.1160662 PMID: 18948539

28. Gentry AH. Changes in plant community diversity and floristic composition on environmental and geographical gradients. Ann Missouri Bot Gard. 1988; 75: 1–34.

29. Baraloto C, Morneau F, Bonal D, Blanc L, Ferry B. Seasonal water stress tolerance and habitat associations within four neotropical tree genera. Ecology. 2007; 88: 478–489. PMID: 17479765

30. Brousseau L, Bonal D, Cigna J, Scotti I. Highly local environmental variability promotes intrapopulation divergence of quantitative traits: an example from tropical rain forest trees. Ann Bot. 2013; 112: 1169–1179. doi: 10.1093/aob/mct176 PMID: 24023042

31. ter Steege H, Pitman NCA, Sabatier D, Baraloto C, Salomão RP, Guevara JE, et al. Hyperdominance in the Amazonian tree flora. Science. 2013; 342.

32. Cowan RS. A monograph of the genus *Eperua* (Leguminosae-Caesalpinioideae): Smithsonian Institution Press; 1975.

33. Frazer GW, Canham CD, Lertzman KP. Gap Light Analyzer (GLA), Version 2.0: Imaging software to extract canopy structure and gap light transmission indices from true-colour fisheye photographs, users manual and program documentation: Simon Fraser University, Burnaby, British Columbia, and the Institute of Ecosystem Studies, Millbrook, New York; 1999.

34. Foll M, Gaggiotti O. A genome-scan method to identify selected loci appropriate for both dominant and codominant markers: A Bayesian perspective. Genetics. 2008; 180: 977–993. doi: 10.1534/genetics.108.092221 PMID: 18780740

35. Antao T, Beaumont MA. Mcheza: a workbench to detect selection using dominant markers. Bioinformatics. 2011.

36. Storz JF. Using genome scans of DNA polymorphism to infer adaptive population divergence. Mol Ecol. 2005; 14: 671–688. PMID: 15723660

37. Vos P, Hogers R, Bleeker M. AFLP: a new technique for DNA fingerprinting. Nucleic Acids Res. 1995; 23: 4407–4414. PMID: 7501463

38. Campbell D, Bernatchez L. Generic scan using AFLP markers as a means to assess the role of directional selection in the divergence of sympatric whitefish ecotypes. Mol Biol Evol. 2004; 21: 945–956. PMID: 15014172

39. Chybicki IJ, Oleksa A, Burczyk J. Increased inbreeding and strong kinship structure in *Taxus baccata* estimated from both AFLP and SSR data. Heredity. 2011; 107: 589–600. doi: 10.1038/hdy.2011.51 PMID: 21712844

40. Dasmahapatra KK, Lacy RC, Amos W. Estimating levels of inbreeding using AFLP markers. Heredity. 2007; 100: 286–295. PMID: 17987055

41. Gagnaire PA, Albert V, Jonsson B, Bernatchez L. Natural selection influences AFLP intraspecific genetic variability and introgression patterns in Atlantic eels. Mol Ecol. 2009; 18: 1678–1691. doi: 10.1111/j.1365-294X.2009.04142.x PMID: 19302349

42. Jump AS, Penuelas J. Extensive spatial genetic structure revealed by AFLP but not SSR molecular markers in the wind-pollinated tree, *Fagus sylvatica*. Mol Ecol. 2007; 16: 925–936. PMID: 17305851

43. Paris M, Despres L. Identifying insecticide resistance genes in mosquito by combining AFLP genome scans and 454 pyrosequencing. Mol Ecol. 2012; 21: 1672–1686. doi: 10.1111/j.1365-294X.2012.05499.x PMID: 22348648

44. Hardy OJ. Estimation of pairwise relatedness between individuals and characterization of isolation-by-distance processes using dominant genetic markers. Mol Ecol. 2003; 12: 1577–1588. PMID: 12755885

45. Meudt HM, Clarke AC. Almost forgotten or latest practice? AFLP applications, analyses and advances. Trends Plant Sci. 2007; 12: 106–117. PMID: 17303467

46. Campbell D, Duchesne P, Bernatchez L. AFLP utility for population assignment studies: analytical investigation and empirical comparison with microsatellites. Mol Ecol. 2003; 12: 1979–1991. PMID: 12803646

47. Arrigo N, Tuszynski J, Ehrich D, Gerdes T, Alvarez N. Evaluating the impact of scoring parameters on the structure of intra-specific genetic variation using RawGeno, an R package for automating AFLP scoring. BMC Bioinformatics. 2009; 10: 33. doi: 10.1186/1471-2105-10-33 PMID: 19171029

48. Bonin A, Ehrich D, Manel S. Statistical analysis of amplified fragment length polymorphism data: a toolbox for molecular ecologists and evolutionists. Mol Ecol. 2007; 16: 3737–3758. PMID: 17850542

49. Crawford LA, Koscinski D, Keyghobadi N. A call for more transparent reporting of error rates: the quality of AFLP data in ecological and evolutionary research. Mol Ecol. 2012; 21: 5911–5917. doi: 10.1111/mec.12069 PMID: 23121160

50. Ley AC, Hardy OJ. Improving AFLP analysis of large-scale patterns of genetic variation—a case study with the Central African lianas *Haumania* spp (Marantaceae) showing interspecific gene flow. Mol Ecol. 2013; 22: 1984–1997. doi: 10.1111/mec.12214 PMID: 23398575

51. Colpaert N, cavers S, Bandou E, Caron H, Gheysen G, Lowe AJ. Sampling tissue for DNA analysis of trees: trunk cambium as an alternative to canopy leaves. Silvae Genet. 2005; 54: 265–269.

52. Doyle JJ, Doyle JL. A rapid DNA isolation procedure from small quantities of fresh leaf tissues. Phytochem Bull. 1987; 19: 11–15.

53. Montemurro C, Pasqualone A, Simeone R, Sabetta W, Blanco A. AFLP molecular markers to identify virgin olive oils from single Italian cultivars. Eur Food Res Technol. 2008; 226: 1439–1444.

54. Chen DH, Ronald PC. A Rapid DNA Minipreparation Method Suitable for AFLP and Other PCR Applications. Plant Mol Biol Report. 1999; 17: 53–57.

55. Barrett BA, Kidwell KK, Fox PN. Comparison of AFLP and pedigree-based genetic diversity assessment methods using wheat cultivars from the Pacific Northwest. Crop Sci. 1998; 38: 1271–1278.

56. Pritchard JK, Stephens M, Donnelly P. Inference of population structure using multilocus genotype data. Genetics. 2000; 155: 945–959. PMID: 10835412

57. Evanno G, Regnaut S, Goudet J. Detecting the number of clusters of individuals using the software structure: a simulation study. Mol Ecol. 2005; 14: 2611–2620. PMID: 15969739

58. Kopelman NM, Mayzel J, Jakobsson M, Rosenberg NA, Mayrose I. CLUMPAK: a program for identifying clustering modes and packaging population structure inferences across K. Submitted.

59. Hardy OJ, Vekemans X. Isolation by distance in a continuous population: reconciliation between spatial autocorrelation analysis and population genetics models. Heredity. 1999; 83: 145–154. PMID: 10469202

60. Hardy OJ, Vekemans X. SPAGeDi: a versatile computer program to analyse spatial genetic structure at the individual or population levels. Mol Ecol Notes. 2002; 2: 618–620.

61. Lunn D, Thomas A, Best N, Spiegelhalter D. WinBUGS—A Bayesian modelling framework: concepts, structure, and extensibility. Stat Comput. 2000; 10: 325–337. PMID: 10902901

62. Gelfand AE, Smith AFM. Sampling-based approaches to calculating marginal densities. J Am Stat Assoc. 1990; 85: 398–409.

63. Excoffier L, Lischer HEL. Arlequin suite ver 3.5: a new series of programs to perform population genetics analyses under Linux and Windows. Mol Ecol Resour. 2010; 10: 564–567. doi: 10.1111/j.1755-0998.2010.02847.x PMID: 21565059

64. Excoffier L, Smouse PE, Quattro JM. Analysis of molecular variance inferred from metric distances among DNA haplotypes: application to human mitochondrial DNA restriction data. Genetics. 1992; 131: 479–491. PMID: 1644282

65. Excoffier L, Hofer T, Foll M. Detecting loci under selection in a hierarchically structured population. Heredity. 2009; 103: 285–298. doi: 10.1038/hdy.2009.74 PMID: 19623208

66. Strimmer K. A unified approach to false discovery rate estimation. BMC Bioinformatics. 2008; 9: 303. doi: 10.1186/1471-2105-9-303 PMID: 18613966

67. Strimmer K. 'fdrtool': a versatile R package for estimating local and tail area-based false discovery rates. Bioinformatics. 2008; 24: 1461–1462. doi: 10.1093/bioinformatics/btn209 PMID: 18441000

68. Hampe A, Masri LE, Petit RJ. Origin of spatial genetic structure in an expanding oak population. Mol Ecol. 2010; 19: 459–471. doi: 10.1111/j.1365-294X.2009.04492.x PMID: 20070522

69. Jump AS, Rico L, Coll M, Penuelas J. Wide variation in spatial genetic structure between natural populations of the European beech (*Fagus sylvatica*) and its implications for SGS comparability. Heredity. 2012; 108: 633–639. doi: 10.1038/hdy.2012.1 PMID: 22354112

70. Leonardi S, Menozzi P. Spatial structure of genetic variability in natural stands of *Fagus sylvatica L*. (beech) in Italy. Heredity. 1996; 77: 359–368.

71. Oddou-Muratorio S, Bontemps A, Klein EK, Chybicki I, Vendramin GG, Suyama Y. Comparison of direct and indirect genetic methods for estimating seed and pollen dispersal in *Fagus sylvatica* and *Fagus crenata*. For Ecol Manage. 2010; 259: 2151–2159.

72. Streiff R, Labbe T, Bacilieri R, Steinkellner H, Glossl J, Kremer A. Within-population genetic structure in *Quercus robur L*. and *Quercus petraea* (Matt.) Liebl. assessed with isozymes and microsatellites. Mol Ecol. 1998; 7: 317–328.

73. Born C, Hardy OJ, Chevalier M-H, Ossari S, Atteke C, Wickings EJ, et al. Small-scale spatial genetic structure in the Central African rainforest tree species *Aucoumea klaineana*: a stepwise approach to infer the impact of limited gene dispersal, population history and habitat fragmentation. Mol Ecol. 2008; 17: 2041–2050. doi: 10.1111/j.1365-294X.2007.03685.x PMID: 18331246

74. Cloutier D, Kanashiro M, Iampil AYC, Schoen DJ. Impact of selective logging on inbreeding and gene dispersal in an Amazonian tree population of *Carapa guianensis* Aubl. Mol Ecol. 2006; 16: 1–13.

75. Collevatti RG, Lima JS, Soares TN, Telles MPdC. Spatial genetic structure and life history traits in Cerrado tree species: Inferences for conservation. Natureza & Conservacao. 2010; 08: 54–59.

76. Dick CW, Etchelecu G, Austerlitz F. Pollen dispersal of tropical trees (*Dinizia excelsa*: Fabaceae) by native insects and African honeybees in pristine and fragmented Amazonian rainforest. Mol Ecol. 2003; 12: 753–764. PMID: 12675830

77. Doligez A, Joly HI. Genetic diversity and spatial structure within a natural stand of a tropical forest tree species, *Carapa procera* (Meliaceae), in French Guiana. Heredity. 1997; 79: 72–82.

78. Konuma A, Tsumura Y, Lee CT, Lee SL, Okuda T. Estimation of gene flow in the tropical-rainforest tree *Neobalanocarpus heimii* (Dipterocarpaceae), inferred from paternity analysis. Mol Ecol. 2000; 9: 1843–1852. PMID: 11091320

79. Lowe AJ, Jourde B, Breyne P, Colpaert N, Navarro C, Wilson J, et al. Fine-scale genetic structure and gene flow within Costa Rican populations of mahogany (*Swietenia macrophylla*). Heredity. 2003; 90: 268–275. PMID: 12634811

80. Cavers S, Degen B, Caron H, Lemes MR, Margis R, Salgueiro F, et al. Optimal sampling strategy for estimation of spatial genetic structure in tree populations. Heredity. 2005; 95: 281–289. PMID: 16030529

81. Dutech C, Seiter J, Petronelli P, Joly HI, Jarne P. Evidence of low gene flow in a neotropical clustered tree species in two rainforest stands of French Guiana. Mol Ecol. 2002; 11: 725–738. PMID: 11972760

82. Latouche-Halé C, Ramboer A, Bandou E, Caron H, Kremer A. Long-distance pollen flow and tolerance to selfing in a neotropical tree species. Mol Ecol. 2004; 13: 1055–1064. PMID: 15078444

83. Heuertz M, Vekemans X, Hausman JF, Palada M, Hardy OJ. Estimating seed vs. pollen dispersal from spatial genetic structure in the common ash. Mol Ecol. 2003; 12: 2483–2495. PMID: 12919486

84. Nosil P, Egan SP, Funk DJ. Heterogeneous genomic differentiation between walking-stick ecotypes: Isolation by adaptation and multiple roles for divergent selection. Evolution. 2008; 62: 316–336. PMID: 17999721

85. Amato R, Pinelli M, Monticelli A, Marino D, Miele G, Cocozza S. Genome-wide scan for signatures of human population differentiation and their relationship with natural selection, functional pathways and diseases. PLoS ONE. 2009; 4: e7927. doi: 10.1371/journal.pone.0007927 PMID: 19936260

86. Burgarella C, Navascuas M, Zabal-Aguirre M, Berganzo E, Riba M, Mayol M, et al. Recent population decline and selection shape diversity of taxol-related genes. Mol Ecol. 2012; 21: 3006–3021. doi: 10.1111/j.1365-294X.2012.05532.x PMID: 22574693

87. Eckert A, Wegrzyn JL, Pande B, Jermstad KD, Lee JM, Liechty JD, et al. Multilocus patterns of nucleotide diversity and divergence reveal positive selection at candidate genes related to cold-hardiness in coastal Douglas-fir (*Pseudotsuga menziesii* var. *menziesii*). Genetics. 2009; 183: 289–298. doi: 10.1534/genetics.109.103895 PMID: 19596906

88. Eckert AJ, Heerwaarden JV, Wegrzyne JL, Nelson CD, Ross-Ibarra J, Gonzalez-Martinez SC, et al. Patterns of population structure and environmental associations to aridity across the range of Loblolly Pine (*Pinus taeda* L., Pinaceae). Genetics. 2010; 185: 969–982. doi: 10.1534/genetics.110.115543 PMID: 20439779

89. Eveno E, Collada C, Guevara MA, Léger V, Soto A, Diaz L, et al. Contrasting patterns of selection at *Pinus pinaster* Ait. drought stress candidate gees as revealed by genetic differenciation analyses. Mol Biol Evol. 2008; 25: 417–437. PMID: 18065486

90. Alberto FJ, Derory J, Boury C, Frigerio J-M, Zimmermann NE, Kremer A. Imprints of natural selection along environmental gradients in phenology-related genes of *Quercus petraea*. Genetics. 2013; 195: 495–512. doi: 10.1534/genetics.113.153783 PMID: 23934884

91. Jump AS, Hunt JM, Martinez-Izquierdo JA, Penuelas J. Natural selection and climate change: temperature-linked spatial and temporal trends in gene frequency in *Fagus sylvatica*. Mol Ecol. 2006; 15: 3469–3480. PMID: 16968284

92. Savolainen V, Anstett M-C, Lexer C, Hutton I, Clarkson JJ, Norup MV, et al. Sympatric speciation in palms on an oceanic island. Nature. 2006; 441: 210–213. PMID: 16467788

93. Beaumont MA, Balding DJ. Identifying adaptive genetic divergence among populations from genome scans. Mol Ecol. 2004; 13: 969–980. PMID: 15012769

94. Le Corre V, Kremer A. The genetic differentiation at quantitative trait loci under local adaptation. Mol Ecol. 2012; 21: 1548–1566. doi: 10.1111/j.1365-294X.2012.05479.x PMID: 22332667

95. Bierne N, Roze D, Welch JJ. Pervasive selection or is it? Why are FST outliers sometimes so frequent? Mol Ecol. 2013; 22: 2061–2064. PMID: 23671920

96. Hermisson J. Who believes in whole-genome scans for selection? Heredity. 2009; 103: 283–284. doi: 10.1038/hdy.2009.101 PMID: 19654610

97. Bonin A, Taberlet P, Miaud C, Pompanon F. Explorative genome scan to detect candidate loci for adaptation along a gradient of altitude in the common frog (*Rana temporaria*). Mol Biol Evol. 2006; 23: 773–783. PMID: 16396915

98. Beaumont MA. Adaptation and speciation: what can Fst tell us? Trends Ecol Evol. 2005; 20: 435–440. PMID: 16701414

99. Nielsen R, Williamson S, Kim Y, Hubisz MJ, Clark AG, Bustamante C. Genomic scans for selective sweeps using SNP data. Genome Res. 2005; 15: 1566–1575. PMID: 16251466

100. Galindo J, Moran P, Rolan-Alvarez E. Comparing geographical genetic differentiation between candidate and noncandidate loci for adaptation strengthens support for parallel ecological divergence in the marine snail *Littorina saxatilis*. Mol Ecol. 2009; 18: 919–930. doi: 10.1111/j.1365-294X.2008.04076.x PMID: 19207246

101. Makinen HS, Cano JM, Merila J. Identifying footprints of directional and balancing selection in marine and freshwater three-spined stickleback (*Gasterosteus aculeatus*) populations. Mol Ecol. 2008; 17: 3565–3582. doi: 10.1111/j.1365-294X.2008.03714.x PMID: 18312551

102. Meier K, Hansen MM, Bekkevold D, Skaala O, Mensberg KLD. An assessment of the spatial scale of local adaptation in brown trout (*Salmo trutta L.*): footprints of selection at microsatellite DNA loci. Heredity. 2011; 106: 488–499. doi: 10.1038/hdy.2010.164 PMID: 21224872

103. Oetjen K, Reusch TBH. Genome scans detect consistent divergent selection among subtidal vs. intertidal populations of the marine angiosperm *Zostera marina*. Mol Ecol. 2007; 16: 5156–5157. PMID: 17986196

104. Pariset L, Joost S, Marsan P, Valentini A. Landscape genomics and biased FST approaches reveal single nucleotide polymorphisms under selection in goat breeds of North-East Mediterranean. BMC Genet. 2009; 10: 7. doi: 10.1186/1471-2156-10-7 PMID: 19228375

105. Prunier J, Laroche J, Beaulieu J, Bousquet J. Scanning the genome for gene SNPs related to climate adaptation and estimating selection at the molecular level in boreal black spruce. Mol Ecol. 2011; 20: 1702–1716. doi: 10.1111/j.1365-294X.2011.05045.x PMID: 21375634

106. Soto-Cerda BJ, Cloutier S. Outlier loci and selection signatures of simple sequence repeats (SSRs) in flax (*Linum usitatissimum L.*). Plant Mol Biol Report. 2013: 1–13.

107. Storz JF, Dubach JM. Natural selection drives altitudinal divergence at the albumin locus in deer mice, *Peromyscus maniculatus*. Evolution. 2004; 58: 1342–1352. PMID: 15266982

108. McKay JK, Latta RG. Adaptive population divergence: markers, QTL and traits. Trends Ecol Evol. 2002; 17: 285–291.

109. Sork VL, Aitken SN, Dyer RJ, Eckert AJ, Legendre P, Neale DB. Putting the landscape into the genomics of trees: approaches for understanding local adaptation and population responses to changing climate. Tree Genet Genomes. 2013; 9: 901–911.
